# MIR22HG acts as a tumor suppressor via TGFβ/SMAD signaling and facilitates immunotherapy in colorectal cancer

**DOI:** 10.1186/s12943-020-01174-w

**Published:** 2020-03-04

**Authors:** Juan Xu, Tingting Shao, Mingxu Song, Yunjin Xie, Jialiang Zhou, Jiaqi Yin, Na Ding, Haozhe Zou, Yongsheng Li, Jiwei Zhang

**Affiliations:** 1 Key Laboratory of Tropical Translational Medicine of Ministry of Education, College of Biomedical Information and Engineering, Hainan Medical University, Haikou, 571199 China; 2 College of Bioinformatics Science and Technology, Harbin Medical University, Harbin, 150081 Heilongjiang China; 3 Wuxi Oncology Institute, The Affiliated Hospital of Jiangnan University, Wuxi, 214062 China; 4 Department of radiation oncology, The Affiliated Hospital of Jiangnan University, Wuxi, 214062 China; 5 The MOE Key Laboratory for Standardization of Chinese Medicines, Institute of Chinese Materia Medica, Shanghai University of Traditional Chinese Medicine, Shanghai, 201203 China

**Keywords:** MIR22HG, TGFβ pathway, Colorectal cancer, Immunotherapy, SMAD2

## Abstract

**Background:**

Long noncoding RNAs (lncRNAs) are emerging as critical regulatory elements and play fundamental roles in the biology of various cancers. However, we are still lack of knowledge about their expression patterns and functions in human colorectal cancer (CRC).

**Methods:**

Differentially expressed lncRNAs in CRC were identified by bioinformatics screen and the level of MIR22HG in CRC and control tissues were determined by qRT-PCR. Cell viability and migration capacities were examined by MTT and transwell assay. Mouse model was used to examine the function and rational immunotherapy of MIR22HG in vivo.

**Results:**

We systematically investigated the expression pattern of lncRNAs and revealed MIR22HG acts as a tumor suppressor in CRC. The expression of MIR22HG was significantly decreased in CRC, which was mainly driven by copy number deletion. Reduced expression of MIR22HG was significantly associated with poor overall survival. Silencing of MIR22HG promoted cell survival, proliferation and tumor metastasis in vitro and in vivo. Mechanistically, MIR22HG exerts its tumor suppressive activity by competitively interacting with SMAD2 and modulating the activity of TGFβ pathway. Decreased MIR22HG promoted the epithelial-mesenchymal transition in CRC. Importantly, we found that MIR22HG expression is significantly correlated with CD8A and overexpression of MIR22HG triggers T cell infiltration, enhancing the clinical benefits of immunotherapy.

**Conclusion:**

MIR22HG acts as a tumor suppressor in CRC. Our data provide mechanistic insights into the regulation of MIR22HG in TGFβ pathway and facilitates immunotherapy in cancer.

## Background

Colorectal cancer (CRC) is one of the most common malignant tumors with increasing incidence worldwide. Despite therapeutic advances over decades, the majority of CRC patients are diagnosed at an advanced stage with poor prognosis [1]. Thus, it is still a challenge to find novel biomarkers for CRC as well as identifying effective therapeutic targets.

With the development of next-generation sequencing technology, it is realized that the most part of human genome is transcribed to noncoding RNAs [2]. Of these newly discovered RNA regulatory elements, long noncoding RNAs (lncRNAs) have been demonstrated to play critical roles in diverse cellular processes [3–5]. LncRNAs can serve as signal mediators, scaffold or molecular decoys to function in epigenetic, transcriptional and post-transcriptional regulation. For example, HOTAIR is found to be associated with poor prognosis in CRC by regulating polycomb-dependent chromatin modification [6]. CCAT1, CCAT2 and CCAT1-L have been shown to function as oncogenes by up-regulating c-Myc, promoting tumor cell proliferation and migration [7–9]. In addition, several lncRNAs play their roles through competing binding sites of microRNAs, such as HULC [10] and CRNDE [11]. LncRNA SNHG1 can regulate CRC cell growth through interactions with EZH2 [12]. Linc00659 acts as an oncogene in regulating cell growth in CRC by impairing cell cycle progression [13]. Despite the growing list of annotated lncRNAs in CRC, the experimentally verified is small overall. The genome-wide expression pattern and function of lncRNAs in CRC still need to be analyzed.

Moreover, immunotherapy is rapidly becoming a novel therapy for many cancers, including CRC [14]. However, some patients had been generally resistant to immunotherapy. To improve the response rates, it is critical to identify more specific biomarkers and immune checkpoint inhibitors. LncRNAs are emerging as critical regulators of gene expression in the immune system and play critical roles in the development and progression of cancer [15, 16]. However, only a few immune-related lncRNAs have been conducted in cancer so far [17–19]. Therefore, further studies on lncRNAs in immune regulation will be essential to identify the immunotherapy targets in cancer.

Here, we have discovered a novel lncRNA-MIR22HG, by analysis of genome-wide transcriptome across > 1500 CRC samples. We illustrated that MIR22HG is significantly downregulated in CRC, which is mainly driven by copy number deletion. Silencing of MIR22HG is associated with poor prognosis in CRC patients. Furthermore, we found that knockdown with shRNAs of MIR22HG significantly promote cell survival, proliferation and tumor metastasis in vitro and in vivo. MIR22HG functions through regulating the TGFβ pathway via competing interacting with SMAD2. Decrease of MIR22HG promotes the epithelial-mesenchymal transition in CRC. Finally, we found that overexpression of MIR22HG can enhance the response of immunotherapy by increasing the CD8 T cell infiltration, suggesting a rational combinational therapy strategy in CRC.

## Methods

### Human patients

We collected 163 CRC patients and their clinical information in China. In total, 79 paired human CRC tissues and normal controls were collected from Affiliated Hospital of Jiangnan University. Another 84 paired cancer and normal tissues were collected at Fudan University Shanghai Cancer Center. There were 21 CRC patients with liver metastasis among these patients. All patient materials were obtained with informed consent and this study was carried out under the permission of the Clinical Research Ethics Committees.

### Cell lines

The CRC cell lines LoVo and HCT116 were purchased from American Type Culture Collection (ATCC) and cultured following the recommended instructions. These cells were characterized by Genewiz Inc. (China) using short tandem repeat (STR) markers and were confirmed to be Mycoplasma-free (last tested in year 2017).

### Genome-wide expression of lincRNAs in CRC

Genome-wide gene expression across 478 colon adenocarcinoma (COAD) patients and 41 normal controls, and 166 rectum adenocarcinoma (READ) patients and 10 normal controls were downloaded from TCGA project [20]. Gene expression was measured by Fragments Per Kilobase of transcript per Million mapped reads (FPKM). Next, we download the gene and lncRNA annotation from GENCODE project [21]. Here, we focused on the long intergenic noncoding RNAs (lincRNAs) and obtained 7520 lincRNAs for further analysis.

In addition, we searched Gene Expression Omnibus (GEO) by using ‘colorectal cancer’ as keywords and obtained nine datasets across 1311 samples (Table 1). The expression of MIR22HG was extracted in our analysis. The difference of MIR22HG expression in cancer and normal was compared by Wilcox rank sum test.

**Table 1:** The genome-wide expression profiles of CRC patients used in this study

Datasets	#GEO	Samples
TCGA-COAD	GDC	478 cancer and 41 normal samples
TCGA-READ	GDC	166 cancer and 10 normal samples
*Reumers* et al.	GSE117606	65 normal, 59 adenoma and 74 tumors
*Kim* et al.	GSE50760	18 normal, 18 tumor and 18 metastasis patients
*Sheffer* et al.	GSE41258	54 normal, 186 tumor, 47 liver and 20 lung metastasis patients
*Marra* et al.	GSE8671	32 normal and 32 tumors
*Uddin* et al.	GSE23878	24 normal and 35 tumors
*Skrzypczak* et al.	GSE20916	34 normal, 45 adenoma and 36 adenocarcinoma
*Matsuyama* et al.	GSE18105	17 normal, 17 tumor and 77 laser-capture microdissection patients
*Smith* et al.	GSE17536	177 patient with overall survival and disease-free survival
*Jorissen* et al.	GSE14333	226 patients with survival time

### Identification of differentially expressed lincRNAs

The expression of lincRNAs was first log transformed. To identify the lincRNAs that are perturbed in colorectal cancer, we used Wilcoxon rank sum test to identify the differentially expressed lincRNAs in TCGA COAD and READ datasets. The *p*-values were adjusted by Benjamini-Hochberg procedure. The lincRNAs with adjusted *p*-values (false discovery rate, FDR) less than 0.005 and fold change (FC) greater than 3-folds were considered as differentially expressed in colorectal cancer.

### Literature curation of lincRNAs

To investigate whether the differentially expressed lincRNAs are involved in cancer, we used lincRNA symbols and ‘cancer’ as keywords to query the PubMed. This process was performed by the R package ‘RISmed’ (https://rdrr.io/cran/RISmed/). The up-regulated and down-regulated lincRNAs are ranked based on the number of literature, separately.

### Gene Set Enrichment Analysis

To identify the pathways potentially regulated by MIR22HG, we first calculated the expression correlation coefficient for each gene with MIR22HG. Here, we analyzed the gene expression from COAD and READ of TCGA, separately [20]. We next ranked all the genes based on the correlation coefficient (R) and these genes are subjected to Gene Set Enrichment Analysis (GSEA) analysis [22, 23]. The pathways with adjusted *p*-values< 0.01 were identified. We further calculated the proportion of leading edges genes in each enriched pathway. The cancer hallmark-related signaling pathways from MsigDB were considered [24].

### Immune-related scores estimation and immune cell deconvolution

We calculated three immune response-related scores estimated from gene expression of CRC patients. The immune scores were estimated based on ESTIMATE [25] and MHC scores were estimated from expression of “core” MHC-I set [26]. A simple and quantitative measure of immune cytolytic activity (‘CYT’) were calculated based on expression levels of granzyme A (GZMA) and perforin (PRF1) [27]. In addition, TIMER was used to estimate the abundances of member cell types in a mixed cell population [28]. The mutation burden of each CRC patient was calculated as non-silent mutations per Mb.

### Statistical analysis

All results are presented as the mean values+SEM. Wilcox rank sum test, student t test, and the Mann-Whitney U test were used to compared the differences among different groups. The Kaplan-Meier method and log-rank test were used to determine the differences in survival rates between MIR22HG low and high expression groups. All statistical analyses were carried out using R 3.5.1. *P* values < 0.05 were considered statistically significant.

### Supporting materials and methods

For details regarding the quantitative RT-PCR, vector construction and siRNA, overexpression and knockdown, cell proliferation, colony formation, cell migration and invasion assays, tumour formation and metastasis assays, RNA pull-down and mass spectrometry, RNA immunoprecipitation, western blot, immunohistochemistry and other related procedures, TGFβ signaling pathway inhibition, refer to the Supporting materials (Additional file 1: Supporting Materials and Methods).

## Results

### Integrative analysis reveals MIR22HG as a tumor suppressor in CRC

**Fig. 1:**
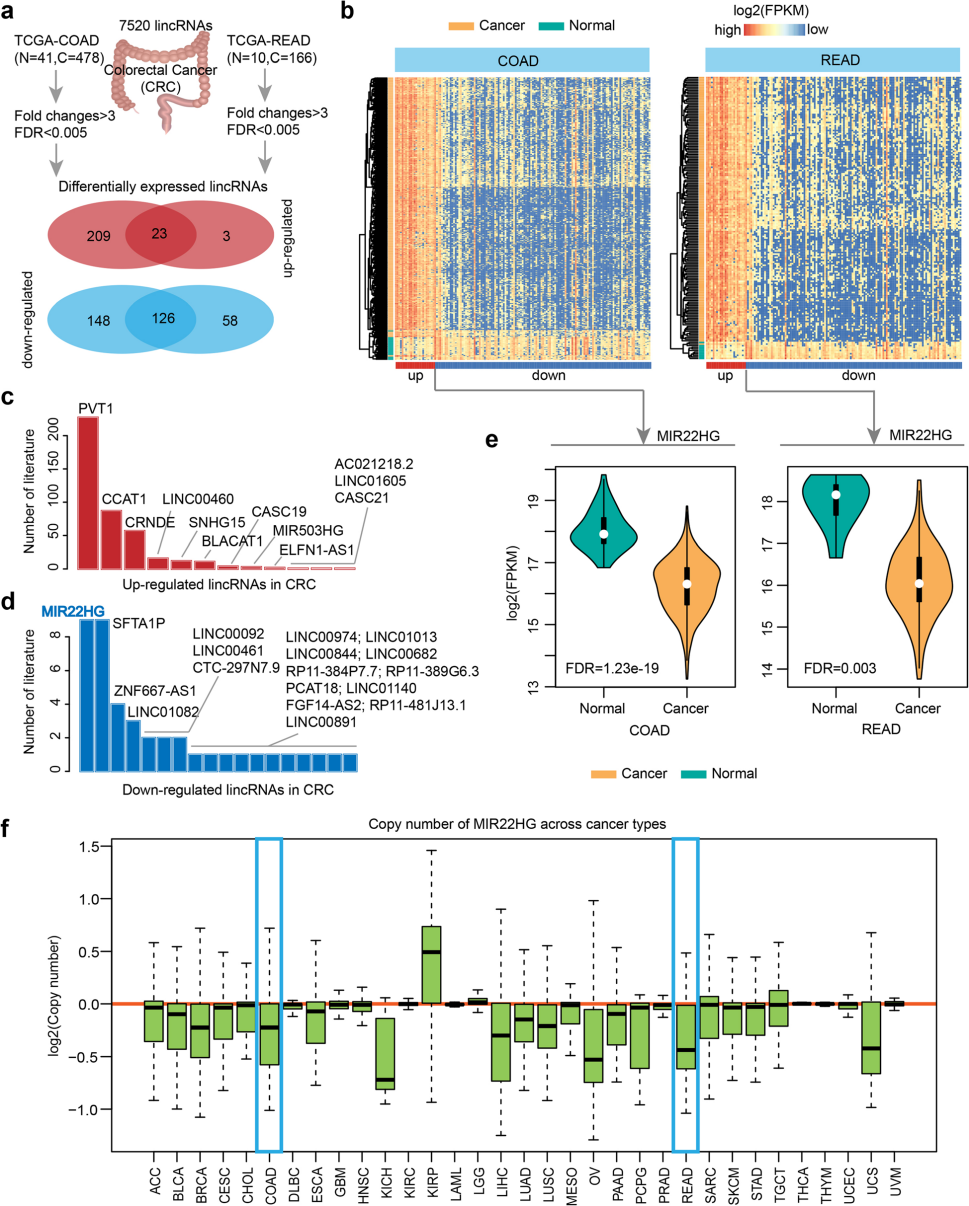
Genome-wide expression analysis of lincRNAs in CRC**. a**, The number of differentially expressed lincRNAs in COAD and READ. The Venn plot in red color shows the up-regulated lincRNAs and the blue ones show the down-regulated lincRNAs in cancer. **b**, Heat maps show the expression of lincRNAs that are differentially expressed both in COAD and READ. **c** and **d**, The number of literature that co-occurred with cancer for each lincRNA. **c** is for up-regulated lincRNAs and **d** is for down-regulated lincRNAs. **e**, The boxplots show the distribution of MIR22HG expression in normal and cancer samples. Left is for COAD and right is for READ. **f**, The log2(copy number) distribution of MIR22HG across cancer types. Blue boxes highlighted the COAD and READ

To identify lncRNAs that paly critical roles in CRC, we first analyzed the genome-wide expression data in COAD and READ of TCGA project (Fig. 1a and Additional file 2: Figure 1a). Particularly, we focused on the 7520 long intergenic noncoding RNAs (lincRNAs). Based on the fold changes > 3-fold and false discovery rate (FDR) < 0.005, we identified 232/26 up-regulated and 274/184 down-regulated lincRNAs in COAD and READ, separately (Fig. 1a). There are 23 up-regulated and 126 down-regulated lincRNAs were identified in both COAD and READ. We found that the expression of these common lincRNAs can effectively distinguish the cancer patients from the normal controls in COAD and READ (Fig. 1b).

Next, we investigated to what extent these lincRNAs were studied in cancer. We found that there are higher number of literature for up-regulated PVT1, CCAT1 and CRNDE (Fig. 1c), suggesting that they have been mostly investigated in cancer. Evidence has demonstrated that PVT1 and CCAT1-encoded lncRNAs have oncogenic functions [29, 30], which is consistent with their higher expression in cancer (Additional file 2: Figure 1b). In the contrast, there is less number of literatures for the down-regulated lincRNAs (Fig. 1d). We found that MIR22HG is the mostly investigated one in cancer and has been show to function as a tumor suppressor in hepatocellular carcinoma [31, 32], lung cancer [33] and thyroid cancer [34]. However, we are still lack of knowledge of its roles in CRC. We found that this lincRNA is not only low expressed in CRC (Fig. 1e, FDR = 1.23E-19 and 0.003), but also shows lower expression in liver and lung cancer (Additional file 2: Figure 1c).

Although these results suggest that MIR22HG might play a tumor-suppressor role in cancer, little is known about the regulation of MIR22HG. We thus next investigated the copy number variation (CNV) of MIR22HG across 33 cancer types [35]. We found that the genomic region of MIR22HG shows prevalent CNV loss across cancer types (Fig. 1f and Additional file 2: Figure 1d). Together, these observations suggest that CNV loss might be the main driver of its lower expression in cancer.

### Validation of MIR22HG in independent cohorts of CRC

**Fig. 2:**
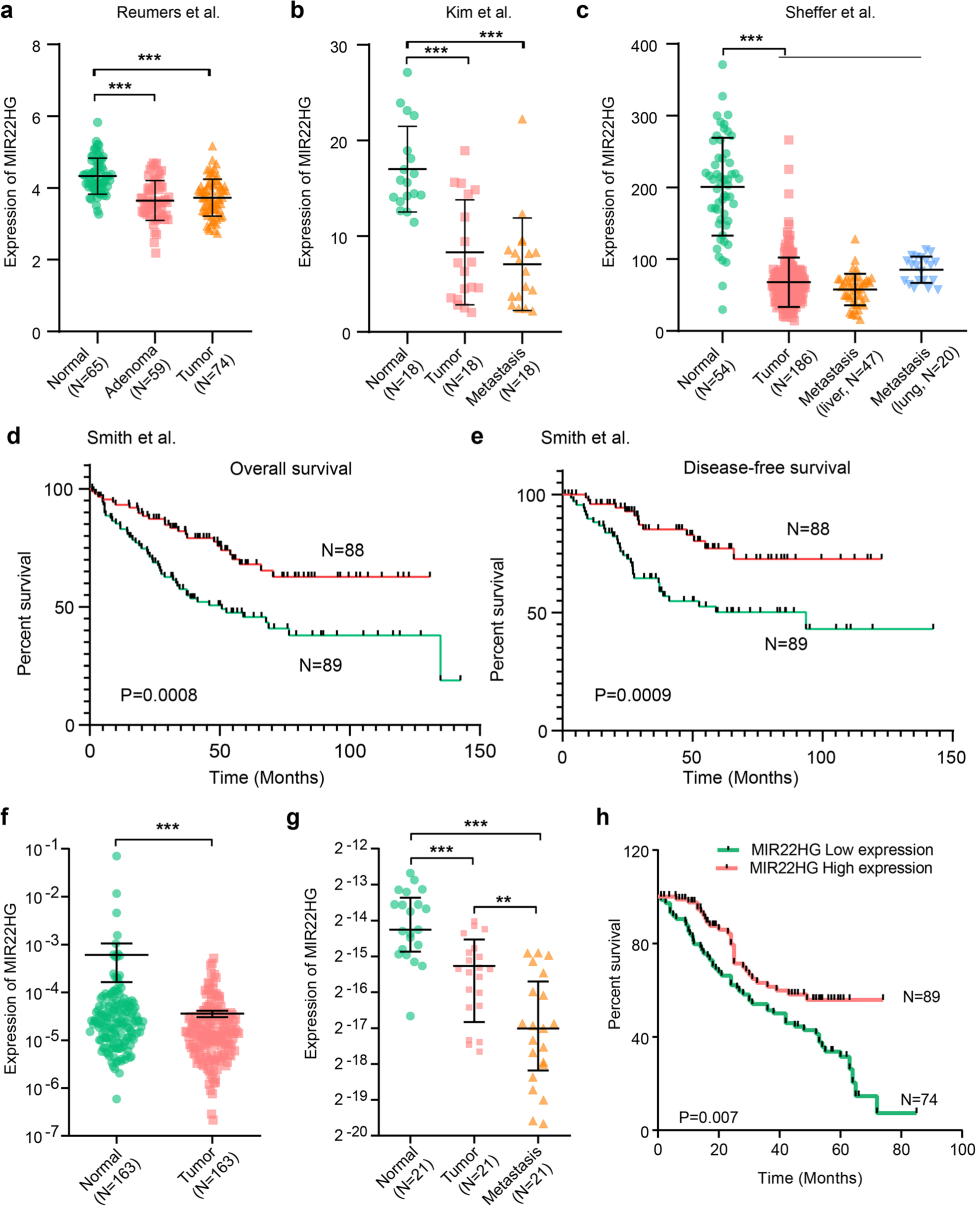
Validation of MIR22HG expression in independent datasets**. a-c**, The distribution of MIR22HG expression in three independent datasets. **d-e**, Kaplan-Meier overall survival curves (**d**) and disease-free survival curves (**e**) are presented. All patients were divided into two groups based on the expression of MIR22HG. Red, high expression of MIR22HG and green, low expression of MIR22HG. **f**, The expression of MIR22HG in 163 normal and tumor samples. **g**, The expression of MIR22HG in 21 normal, tumor and metastasis Chinese samples. **h**, Kaplan-Meier overall survival of Chinese CRC patients based on expression of MIR22HG

To further verify the MIR22HG expression pattern in CRC, we next surveyed the public CRC datasets across 1311 samples. We found that MIR22HG expression levels were significantly lower in CRC as compared with colon tissues in four independent datasets (Fig. 2a and Additional file 2: Figure 2a-d, *p*-values< 0.01). Interestingly, we found that metastatic cancer patients have even lower levels of MIR22HG as compared with primary cancer (Fig. 2b-c). These results indicated that MIR22HG expression is generally lower in CRC and may also be an indicator of metastasis of CRC. Moreover, we investigated the correlation of MIR22HG expression with the patients’ survival. Kaplan-Meier survival curve and log-rank test suggest that lower expression of MIR22HG was significantly associated with poor overall survival and disease-free survival of patients (Fig. 2d-e and Additional file 2: Figure 2e, *p* = 0.0008 and 0.0009).

In addition, we collected 163 CRC patients and used qRT-PCR to investigate the expression of MIR22HG. We found that MIR22HG was significantly low expressed in cancer as compared with normal controls (Fig. 2f, *p* < 0.001). Patients with larger tumor size were significantly enriched in the MIR2HG low expression group (Additional file 3: Table S1). Particularly, liver metastasis patients were also with lower expression of MIR22HG as compared with their own primary cancer (Fig. 2g, *p* < 0.001). Finally, we explored the effects of MIR22HG on prognosis of CRC patients and revealed that lower expression of MIR22HG is significantly associated with poor survival of CRC (Fig. 2h, *p* = 0.007). All these data demonstrated that down-regulation of MIR22HG in CRC may play a tumor suppressor role and could serve as a novel prognostic marker.

### Silencing MIR22HG promotes CRC growth and metastasis in vitro

To investigate the possible function of MIR22HG in CRC pathogenesis, we firstly explored the cellular localization and coding potential of MIR22HG. The MIR22HG expression in both nuclear and cytoplasmic fractions from LoVo and HCT116 were measured by qRT-PCR. We observed that the expression of MIR22HG were in both cytoplasm and nuclear (Additional file 2: Figure 3a-b), suggesting that it may play a regulatory function in both localizations. In addition, we found that MIR22HG didn’t express to protein but play its roles in RNA (Additional file 2: Figure 3c).

**Fig. 3:**
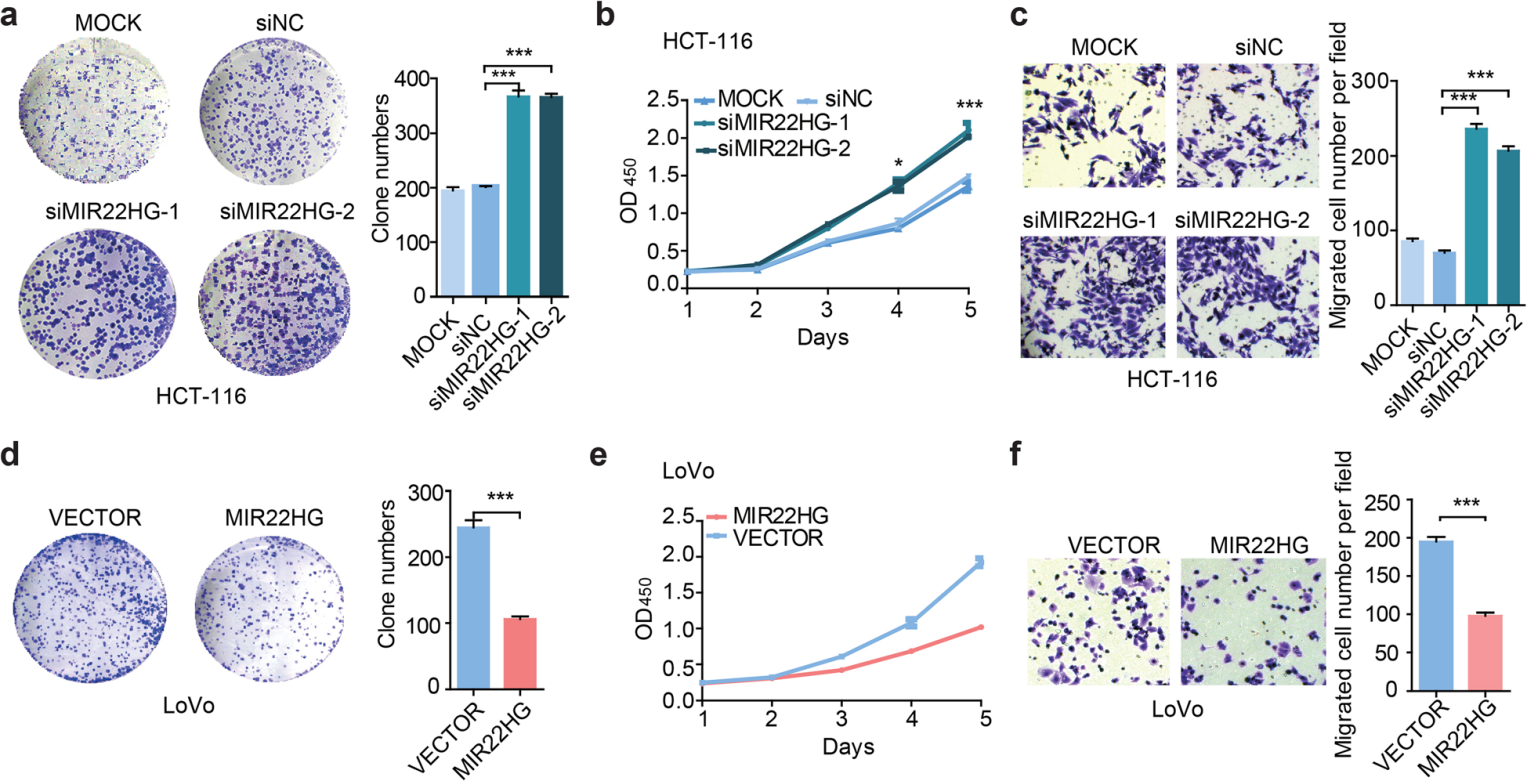
Silencing MIR22HG promotes colorectal cancer cell proliferation and metastasis in vitro. **a**, Representative images and bar graphs showing the effects of MIR22HG knockdown on colony formation in HCT116. **b**, Effects of MIR22HG knockdown on colorectal cancer cell proliferation were measured by CCK-8 assays. **c**, Representative images and bar graphs depicting the migration and invasion abilities of MIR22HG–silenced HCT116 cells. **d-f**, Effects of MIR22HG overexpression on colony formation (**d**), cell proliferation (**e**) and migration (**f**) in LoVo

We next used siRNA technology to knock down the expression of MIR22HG in HCT116 cell line and measured the colony formation, cell proliferation and migration. We found that the colony formation was increased by two-folds as compared with control siRNA (Fig. 3a, *p* < 0.001). Moreover, we further confirmed that cell proliferation was affected by MIR22HG knockdown and there are approximate 70% increase at 96 to 120 h (Fig. 3b, *p* < 0.05). Transwell assays were employed to explore whether cell migration were affected after silencing of MIR22HG. We found that MIR22HG knockdown significantly increased cell migration as compared with control (Fig. 3c, *p* < 0.001).

Moreover, we over-expressed MIR22HG in LoVo and further measured the colony formation, cell proliferation and migration. We found that overexpression of MIR22HG will significantly decrease the number of colony formation (Fig. 3d, *p* < 0.001). Overexpression of MIR22HG also significantly decreased the cell proliferation capability (Fig. 3e) and cell migration (Fig. 3f, *p* < 0.001). Taken together, these results suggested that MIR22HG plays tumor suppressor role by affecting colony formation, proliferation and cell migration.

### Overexpression of MIR22HG suppresses cell proliferation and metastasis in vivo

**Fig. 4:**
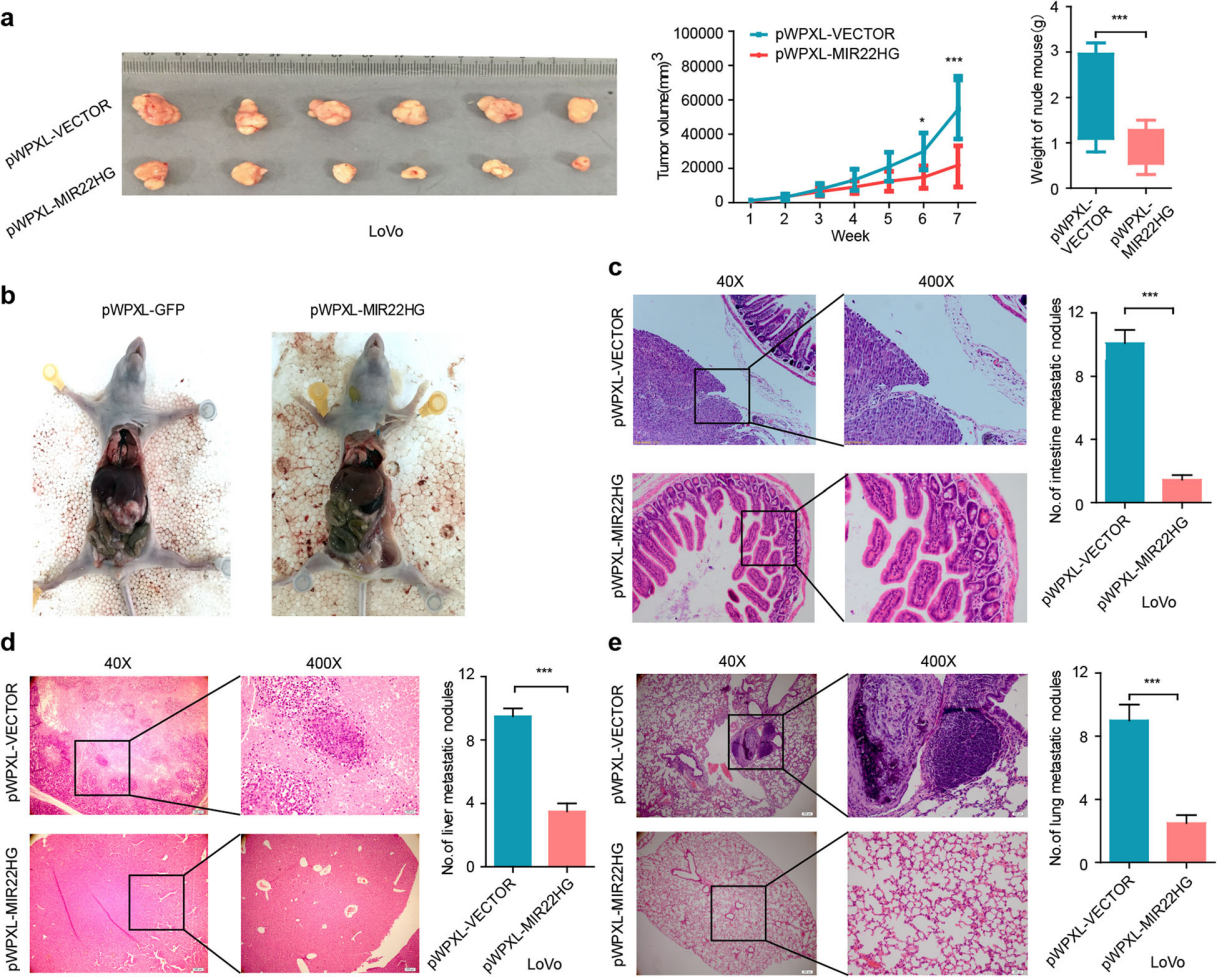
Silencing MIR22HG promotes colorectal cancer cell proliferation and metastasis in vivo**. a**, Images, line and bar graphs showing the effect of MIR22HG overexpression on tumor size, volume and weight of mouse. **b**, Representative images depicting the tumor size of MIR22HG overexpression. **c**, Effect of MIR22HG overexpression in intestine metastatic model. **d**, Effect of MIR22HG on colorectal cancer hepatic metastasis was evaluated using an orthotopic mouse model. **e**, Effect of MIR22HG on tumor metastasis in a lung metastasis mouse model

Furthermore, we examined the effect of MIR22HG overexpression on tumor growth in vivo using mouse subcutaneous xenograft models. We found that tumor xenografts derived from MIR22HG-overexpressing cells exhibited smaller volumes and lower weights than those from empty vector-transduced cells (Fig. 4a, *p *< 0.001). Additionally, we assessed the effects of MIR22HG on colorectal cancer metastasis. Three metastasis models were considered, including an intestine metastasis, a lung metastasis mouse model and an orthotopic hepatic metastasis mouse model. We found that the number of metastatic nodules are significantly reduced in MIR22HG-overexpressing groups (Fig. 4b-e, *p*-values< 0.001). In addition, multiple metastases of different sizes were found in the control mouse, whereas metastatic foci were sparse and small in MIR22HG-overexpressing group. Collectively, these data indicated that overexpression of MIR22HG repressed CRC cell invasion and metastasis.

### MIR22HG perturbs the TGFβ/SMADs signaling pathway in CRC

**Fig. 5:**
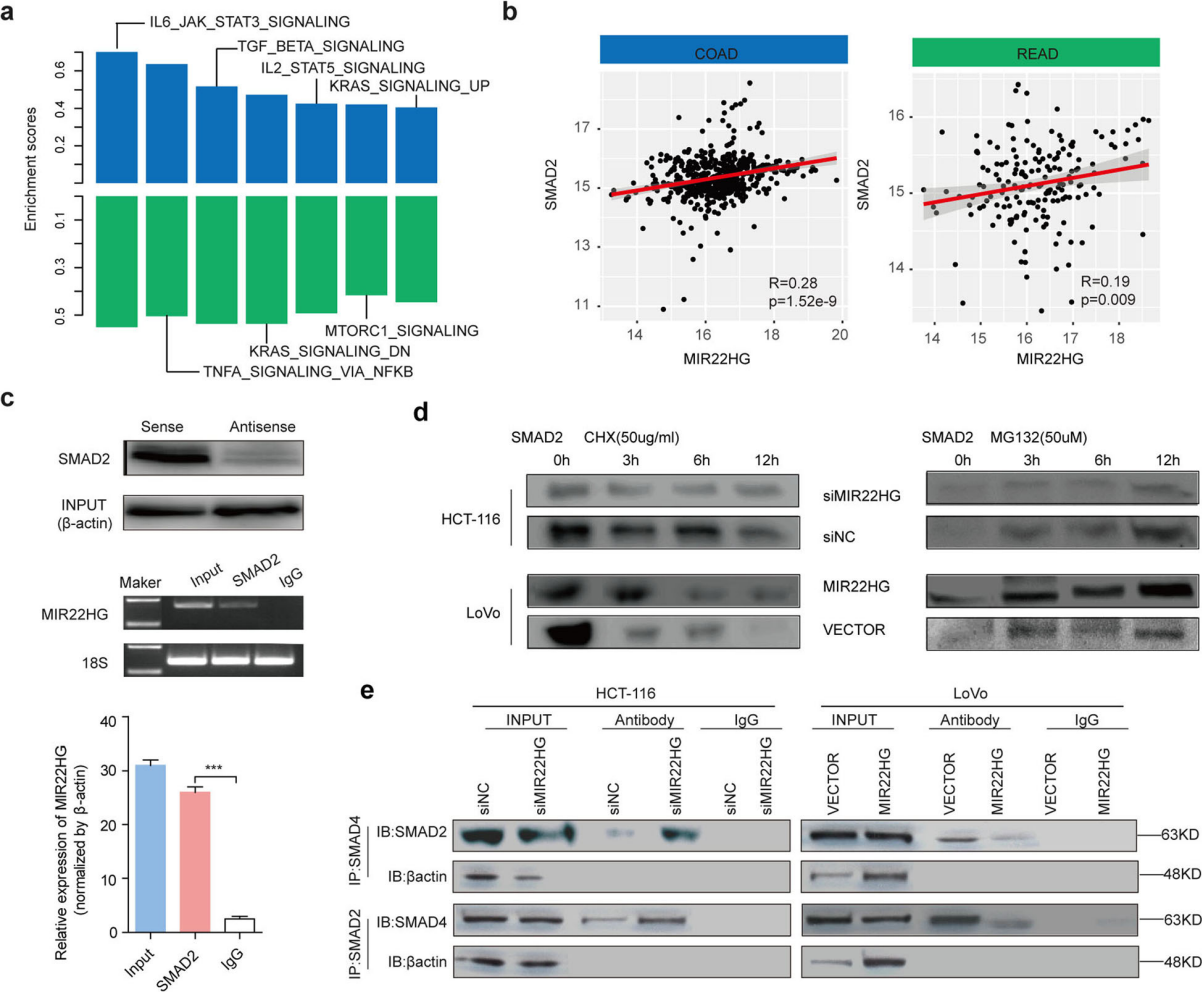
MIR22HG function in TGFβ pathway via competing interaction with SMAD2. **a**, Enriched signaling pathways by genes co-expressed with MIR22HG in COAD and READ. **b**, The scatter plots showing correlation between expression of MIR22HG and SMAD2 in COAD and READ. **c**, Top panels showing the RNA immunoprecipitation between SMAD2 and MIR22HG. Bottom barplot showing the relative expression of MIR22HG in anti-SMAD2 antibodies and IgG. **d**, Immunoblotting for SMAD2 levels in cells treated with over-expressing MIR22HG or shMIR22HG and cycloheximide for different times. **e**, Interaction between SMAD2 and SMAD4 in HCT116 cells transfected with MIR22HG siRNA or in LoVo cells infected with the lentivirus expressing MIR22HG

To elucidate the potential molecular mechanisms of MIR22HG in CRC, we performed the Gene Set Enrichment Analysis (GSEA). We found that the co-expressed genes of MIR22HG are significantly enriched in number of cancer-related signaling pathways (Fig. 5a), including IL6-JAK-STAT3, TNFA signaling and TGFβ signaling pathway. TGFβ signaling plays a pivotal role in diverse cellular processes, such as cell proliferation and migration [36]. We thus investigated the function of MIR22HG in the context of TGFβ signaling pathway. First, we calculated the expression correlation coefficient between MIR22HG and genes in TGFβ pathway. We found that the expression of MIR22HG is significantly correlated with SMAD genes, such as SMAD2 (Fig. 5b), SMAD3 and SMAD4 in both COAD and READ (Additional file 2: Figure 4a). Moreover, the expression of SMAD2 is correlated with patient survival (Additional file 2: Figure 4b). Defects in SMAD signaling can result in TGFβ resistance, causing dysregulation of cell growth [37]. These results suggest that MIR22HG might perform its function via TGFβ/SMAD signaling pathway.

Next, we performed biotin-labeled RNA pull down accompanied by mass spectrometric assays to identify MIR22HG-interacting proteins. The results showed that SMAD2 protein bands at 58.8 kDa in the MIR22HG pull-downed sample (Additional file 2: Figure 4c). We also observed that sense but not antisense of MIR22HG, specifically interact with SMAD2 (Additional file 2: Figure 4c). The interactions were further confirmed through RNA immunoprecipitation (RIP) assays (Fig. 5c). Furthermore, we found that the fragment (674–805 nucleotides) of MIR22HG was responsible for the interaction with SMAD2 (Additional file 2: Figure 4d). MIR22HG knockdown decreased the half-life of SMAD2 protein, whereas ectopic MIR22HG expression increased the half-life of SMAD2 protein in CRC cells (Fig. 5d). Together, these results suggest that MIR22HG can bind to SMAD2 in CRC.

We next characterized the molecular consequences of the associations among MIR22HG and SMAD2. Several lines of evidence have shown that disrupt TGFβ signaling by preventing heteromeric interactions between Smads [38]. Moreover, we found that MIR22HG exerts its biological function by inhibiting the growth and migration of colorectal cancer cells through SMAD2 and TGFβ signaling pathway. Interfering the expression of SMAD2 or the activity of TGFβ signaling pathway were likely to affect the MIR22HG effects on cell growth (Additional file 2: Figure 5a) and cell migration (Additional file 2: Figure 5b). Thus, we hypothesized that MIR22HG might play its function by perturbing the interaction between SMAD2 and SMAD4 by competitively interacting with SMAD2. To test this hypothesis, the interaction between SMAD2 and SMAD4 was examined by co-immunoprecipitation assays, in the presence and absence of MIR22HG. MIR22HG knockdown could enhance the interaction between SMAD2 and SMAD4 (Fig. 5e, left). When MIR22HG was overexpressed, there was less SMAD2 that interacted with SMAD4, as well as less SMAD4 that bound to SMAD2 (Fig. 5e, right), indicating that MIR22HG exhibits a negative regulatory effect on the interaction between SMAD2 and SMAD4. Collectively, these results demonstrated that MIR22HG exerted its tumor suppressive function through competitively binding to SMAD2, thereby preventing the interaction between SMAD2 and SMAD4 in CRC cells.

### Abrogation of MIR22HG promotes epithelial–mesenchymal transition in CRC

**Fig. 6:**
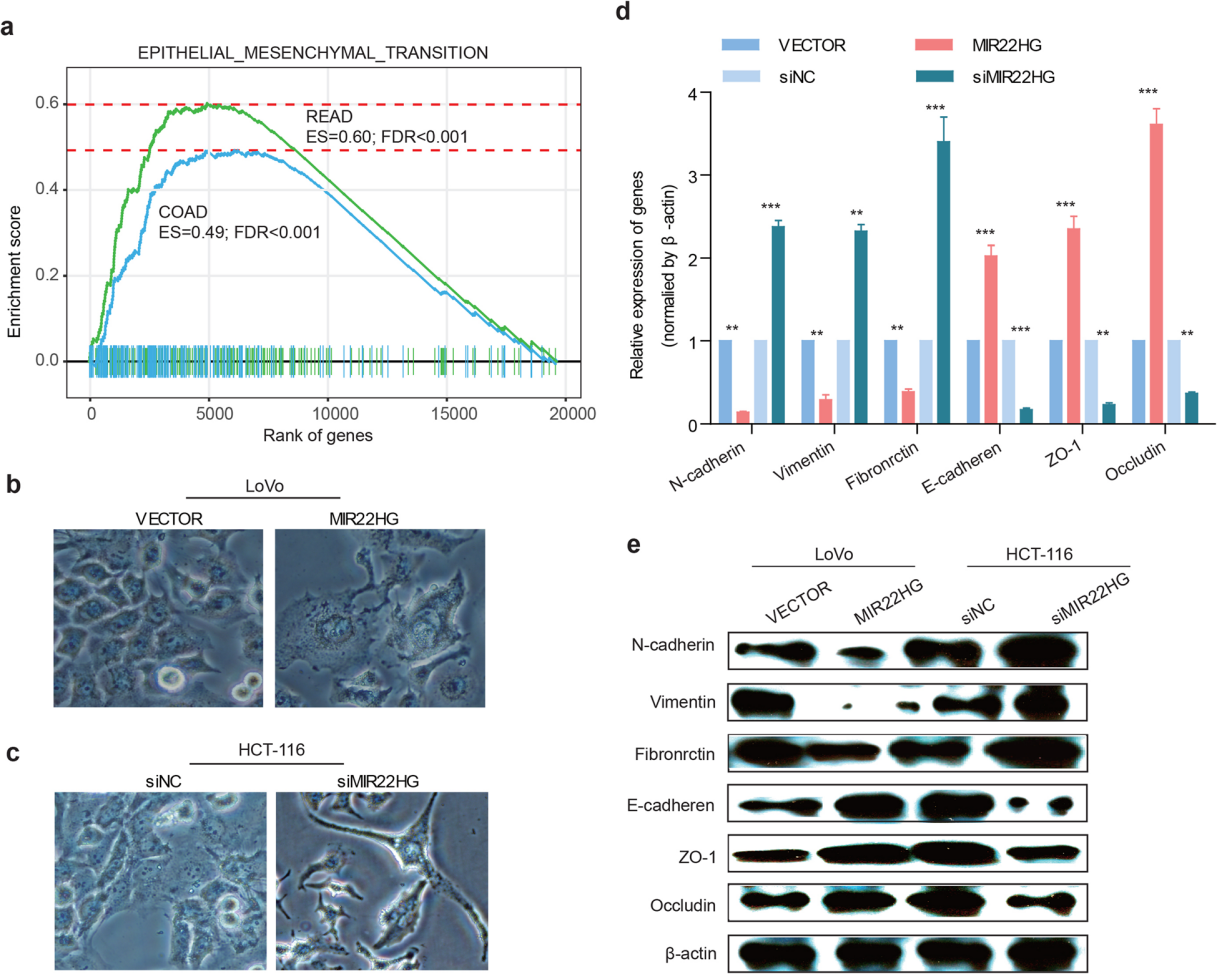
Silencing MIR22HG promoters EMT in CRC. **a**, Enrichment plot of EMT gene sets identified by genes coexpressed with MIR22HG. Blue line representing COAD and green line for READ, FDRs < 0.001. **b** and **c**, LoVo and HCT116 cells overexpressing lncRNA-MIR22HG or siMIR22HG were analyzed using bright-field microscopy. **d**, The expression of EMT relevant markers as determined by qRT-PCR. **e**, Western blot showing the protein level of EMT relevant markers

TGF-beta signaling has been shown to play an important role in epithelial to mesenchymal transition (EMT) [39]. We thus investigated whether MIR22HG plays roles in EMT. Based on GSEA analysis, we found that the co-expressed genes with MIR22HG were significantly enriched in EMT pathway in both COAD and READ (Fig. 6a, FDRs < 0.001). Consistent with these results, we found that the overexpression of MIR22HG led to an epithelial cells property while knockdown MIR22HG acquired mesenchymal properties (Fig. 6b-c). Meanwhile, qRT-PCR and western blot assays showed that the mRNA and protein levels of the epithelial markers (E-cadherin, ZO-1 and Occludin) were markedly decreased and accompanied by an increase in the mesenchymal marker N-cadherin, vimentin and fibronrctin when knockdown MIR22HG (Fig. 6d-e). Moreover, we found that the MIR22HG effects were potentially dependent on the expression of SMAD2 and TGFβ signaling pathway activity. Interfering the expression of SMAD2 and TGFβ signaling pathway inhibition can to a certain extent reverse the EMT gene and protein expression (Additional file 2: Figure 6). These data suggest that the silencing of MIR22HG promotes tumorigenicity in CRC cells by promoting EMT process.

Given that SNAI1 is a transcription factor important for the early step of EMT and SMAD pathway [40, 41], we investigated whether expression of SNAI1 is correlated with SMAD2/SMAD4. We found that SMAD2/SMAD4 can bind to the promoter of SNAI1 (Additional file 2: Figure 7a) and the expressions of SMAD4 are significantly correlated with SNAI1 in CRC (Additional file 2: Figure 7b). Moreover, the expression of SNAI1 is negatively correlated with CDH1 (E-cadherin) and positively correlated with CDH2 (N-cadherin) (Additional file 2: Figure 7c-d). Together, these results suggest that MIR22HG-SMAD2/4-SNAI1 axis play critical roles in CRC by promoting the EMT.

**Fig. 7:**
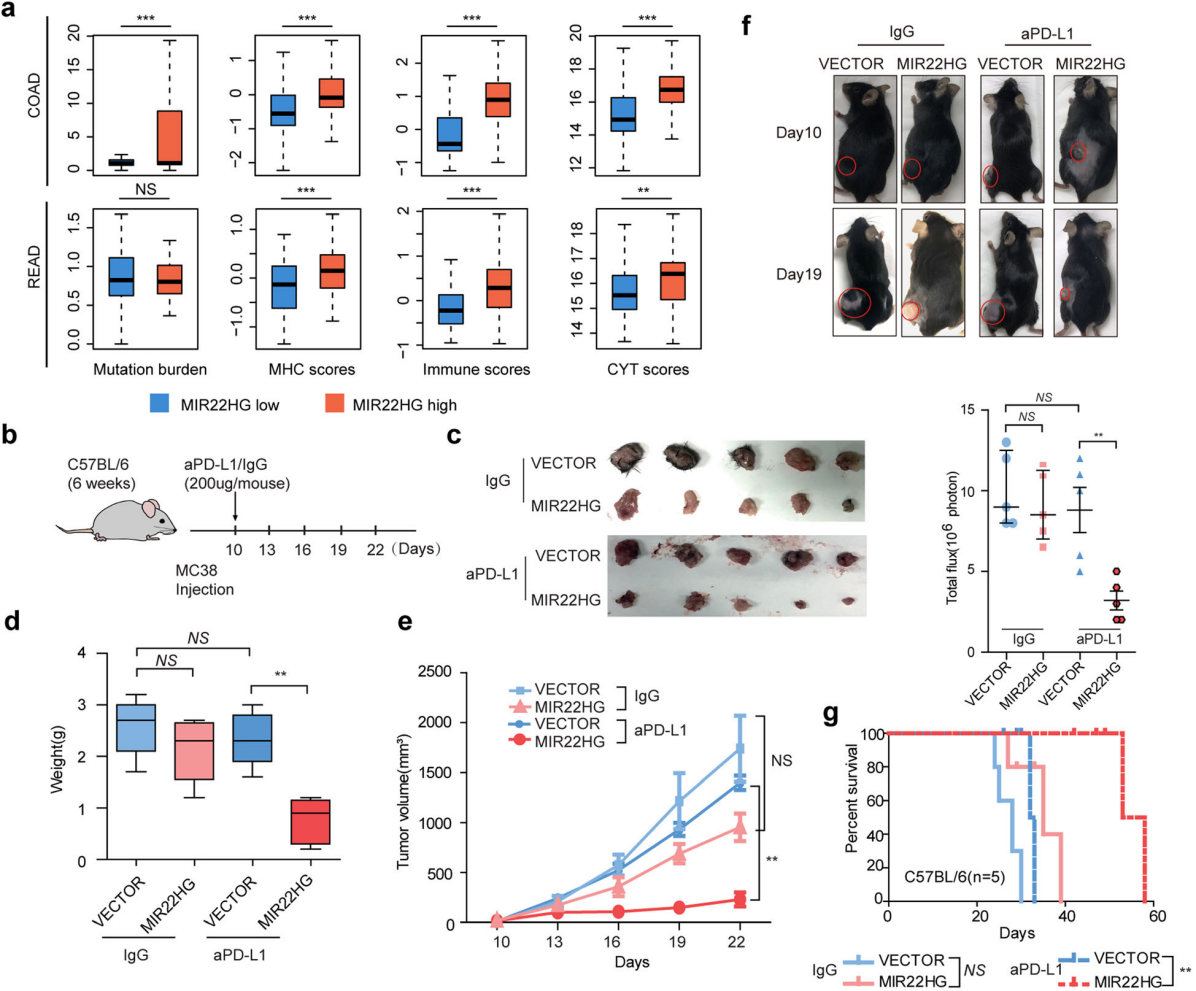
Rational combinational therapy of MIR22HG and aPD-L1 in CRC. **a**, Box plots showing the distribution of mutation burden, MHC, immune and CYT scores in patients with MIR22HG low and high expression. **b**, C57B/6 mice were orthotopically xenografted with MC38 injection and treated with aPD-L1 or IgG every 3 days. **c**, Representative images of tumors treated with IgG + MIR22HG or aPD-L1 + MIR22HG. **d** and **e**, The tumor weight and volume of mice treated with IgG + MIR22HG or aPD-L1 + MIR22HG. **f**, The upper panel showing tumor images of C57B/6 mice. Bottom panel shows the total flux of mice after injections in different groups. **g**, Kaplan-Meier survival curve of C57B/6 mice is shown

### Rational combination immunotherapy of MIR22HG in CRC

Patients with immunogenicity seems to be a promising strategy to develop new drugs that target the antitumor immune response in CRC [42]. However, some patients exhibit primary or acquired resistance. It is critical to identify the additional targets for improving the sensitivity of immunotherapy. Increasing studies have demonstrated that TGFβ pathway affect the expression of PD-L1 and CD8A [43, 44]. We found that the CRC patients with MIR22HG high expression exhibit significantly higher mutation burden, MHC scores, immune scores and CYT scores (Fig. 7a). Moreover, we curated the drug treatment information from TCGA and found that patients with higher MIR22HG expression were likely to response for therapy (Additional file 2: Figure 8). These observations suggest that patients with high MIR22HG expression may be more sensitive to immunotherapy. To further investigate the function of MIR22HG in immune, syngeneic immunocompetent mouse model C57BL/6 mice was used (Fig. 7b). Ten days after CRC implantation, the mice were treated MIR22HG and PD-L1 blockade or IgG. We found that administration of MIR22HG and PD-L1 blockade can inhibit tumor growth (Fig. 7c) and reduce the tumor weight (Fig. 7d, *p* < 0.05). In addition, the mice treated with the combination of MIR22HG and aPD-L1 was with a much smaller tumor volume than the other mice (Fig. 7e). Bioluminescent imaging revealed that overexpression of MIR22HG efficiently enhanced the sensitivity of aPD-L1 treatment (Fig. 7f). The mice receiving a combined treatment demonstrated a slower flux (Fig. 7f) and had a significantly prolonged lifespan (Fig. 7g). Overall, these data demonstrated that MIR22HG serves as a potential therapeutic target to overcome aPD-L1 resistance, enhancing the benefits of aPD-L1 therapy.

**Fig. 8:**
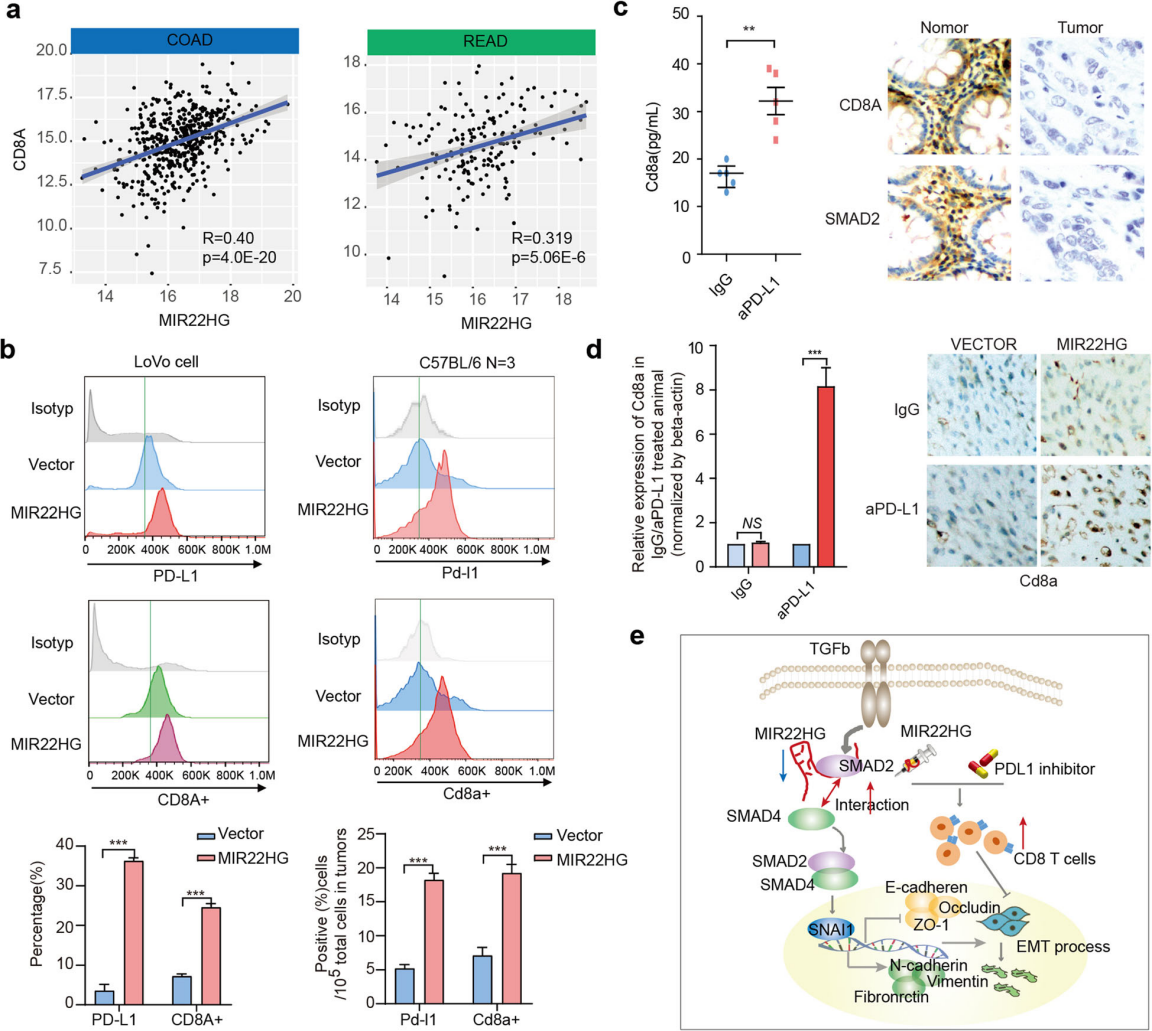
MIR22HG increases the CD8 T cells in CRC. **a**, Scatter plots showing the correlation between CD8A expression and MIR22HG expression. **b**, Cell-surface expression of PD-L1 and CD8A with overexpressing MIR22HG. Left panels for human cell line and right panels for mouse. **c**, Relative expression of Cd8a in mice treated with aPD-L1. The right panels showing the IHC staining of CD8A and SMAD2 in tumor and normal tissues of CRC. **d**, Left panel showing the relative expression of Cd8a in IgG/aPD-L1 + MIR22HG treated mice. Right panel showing the IHC staining of Cd8a. **e**, The mechanistic scheme of lncRNA MIR22HG in CRC

### Overexpression of MIR22HG increases the infiltration of CD8 T cells

We next investigate the potential mechanism for the combination therapy of MIR22HG and aPD-L1. Evidence has indicated that the immune cell infiltration level is correlated with immune therapy response. We thus investigated the expression of MIR22HG with the CD8 T cell infiltration. We found that the expression of MIR22HG is significantly correlated with T cell infiltration in COAD and READ (Additional file 2: Figure 9, *p*-values < 0.05). In addition, the expression of MIR22HG is significantly correlated with the T cell marker gene-CD8A (Fig. 8a). Flow cytometry analysis also shows that overexpressing MIR22HG significantly increases cell-surface expression of PD-L1 and CD8A in vitro and in vivo (Fig. 8b). Moreover, we found that the effects of MIR22HG on CD8A and PD-L1 can be affected by perturbation of SMAD2 expression and TGFβ signaling pathway activity (Additional file 2: Figure 10). These observations suggest that overexpression of MIR22HG is likely to increase the infiltration of CD8 T cells.

To further validate these results, we explored the expression of Cd8a in aPD-L1 treated mice and CRC patient. We found that aPD-L1 treated mice showed increased level of Cd8a (Fig. 8c) and the adjacent normal tissue of CRC patient also showed higher expression of CD8A and SMAD2 (Fig. 8c). These data demonstrated that increase of CD8A is likely to enhance aPD-L1 therapy. We next investigated the expression of Cd8a in mice treated with MIR22HG and aPD-L1. We found that the mice with combined treatment showed increased level of Cd8a than the other mice (Fig. 8d, *p* < 0.001). Taken together, these results demonstrate that overexpression of MIR22HG will increase the infiltration of CD8 T cells in CRC, further enhancing the sensitivity to immune therapy.

## Discussion

With the advancement of high-throughput sequencing technology, increasing lncRNAs have been identified in cancer. LncRNAs have been identified to play critical regulatory function in diverse processes including cancer initiation and progression. In this study, we analyzed the genome-wide lncRNAs expression across > 1500 samples and found the consistent results showing low expression of MIR22HG in CRC. Functional analyses of MIR22HG suggest that silencing of MIR22HG promotes the interactions between SMAD2 and SMAD4 of TGFβ pathway. The SMAD complex binds to the promoter of SNAI1 and further promotes the EMT process (Fig. 8e). Increasing studies have demonstrated that TGFβ pathway can remodel tumor immune environment. Importantly, the expression of MIR22HG is significantly correlated with CD8 T cell infiltration and combination of MIR22HG with aPD-L1 will enhance the sensitivity to immune therapy (Fig. 8e). These results suggest that MIR22HG acts as a tumor suppressor and facilitates immunotherapy in CRC.

By analysis of RNA sequencing data, we found that MIR22HG is low expressed in CRC. To explore the potential mechanism of its low expression, we investigated the CNV alterations of MIR22HG and found that silence of MIR2HG might be driven by CNV deletion. However, some other regulatory issues (such as transcription factors or miRNAs) still need to be addressed for investigate the detail mechanism. In addition, the mechanisms of action of lncRNAs are diverse and include RNA-RNA, RNA-DNA, and RNA-protein interactions [45]. Evidence has shown that MIR22HG is a target of miR-141-3p in endometrial carcinoma [46]. MIR22HG was shown to competing with miRNAs with H19 and play important regulatory roles in breast cancer progression [47]. MIR22HG can also competitively bind to human antigen R (HuR), resulting in weakened expression of HuR-stabilized oncogenes, such as β-catenin in hepatocellular carcinoma cells [32]. MIR22HG was found to inhibit growth, migration and invasion through regulating the miR-10a-5p/NCOR2 axis in hepatocellular carcinoma cells [31]. Silencing of MIR22HG can also triggers cell survival/death signaling via oncogenes YBX1, MET, and p21 in lung cancer [33]. These results suggest the same lncRNA can play diverse roles by regulating different signaling pathways in different tumor context.

It is increasingly clear that there are widespread changes in the expression of lncRNAs during the activation of immune response [48]. A number of lncRNAs have been found to play important roles in immunotherapy. NEAT1 knockdown can induce immune tolerance in vivo and may be a promising therapeutic target in the treatment of immune-related diseases [49]. UCA1 overexpression had been shown to protect PDL1 expression from the repression of miRNAs and contributed to the gastric cancer cells immune escape [50]. Oncogenic LINK-A can downregulate cancer cell antigen presentation and intrinsic tumor suppression in human triple-negative breast cancer [18]. However, limited lncRNAs are found to play immunology function in CRC. We found MIR22HG overexpression triggers CD8 T cell infiltration, suggesting that MIR22HG might be a potential target for immunotherapy in CRC.

## Conclusions

In conclusion, our findings broaden understanding of the function of lncRNAs in CRC. Moreover, our results demonstrate that tumor suppressor MIR22HG may act as a clinical biomarker and overexpression of MIR22HG may be a novel synergistic strategy for enhancing immunotherapy sensitivity, thereby enhancing its clinical benefits for CRC patients.

## Supplementary information

**Additional file 1.** Supporting Materials and Methods.

**Additional file 2.** Supplementary figures, including supplementary Figs S1-S10. 

**Additional file 3. Table S1.** Correlation of the expression of MIR22HG in colorectal cancer with clinic pathologic features.

## Data Availability

The gene expression profiles and clinical data can be found at the GDC portal (https://portal.gdc.cancer.gov/) and GEO (https://www.ncbi.nlm.nih.gov/geo/). The copy number data were downloaded from Broad GDAC Firehose (https://gdac.broadinstitute.org/). Software and resources used for the analyses are described in each method section.

## Abbreviations

CCAT1: Colon Cancer Associated Transcript 1
CNV: Copy number variation
COAD: Colon adenocarcinoma
CRC: Colorectal cancer
CRNDE: Colorectal Neoplasia Differentially Expressed
EMT: Epithelial–mesenchymal transition
FDR: False discovery rate
GSEA: Gene Set Enrichment Analysis
LncRNA: Long non-coding RNA
MIR22HG: MIR22 Host Gene
PD-L1: Programmed death-ligand 1
PVT1: Pvt1 Oncogene
READ: Rectum Adenocarcinoma
shRNA: Short hairpin RNA or small hairpin RNA
SMAD2: SMAD Family Member 2
SMAD4: SMAD Family Member 4
SNAI1: Snail Family Transcriptional Repressor 1
