# Correction: MIR22HG acts as a tumor suppressor via TGFβ/SMAD signaling and facilitates immunotherapy in colorectal cancer

**DOI:** 10.1186/s12943-023-01821-y

**Published:** 2023-07-20

**Authors:** Juan Xu, Tingting Shao, Mingxu Song, Yunjin Xie, Jialiang Zhou, Jiaqi Yin, Na Ding, Haozhe Zou, Yongsheng Li, Jiwei Zhang

**Affiliations:** 1 Key Laboratory of Tropical Translational Medicine of Ministry of Education, College of Biomedical Information and Engineering, Hainan Medical University, Haikou, 571199 China; 2 College of Bioinformatics Science and Technology, Harbin Medical University, Harbin, Heilongjiang 150081 China; 3 Wuxi Oncology Institute, The Affiliated Hospital of Jiangnan University, Wuxi, 214062 China; 4 Department of radiation oncology, The Affiliated Hospital of Jiangnan University, Wuxi, 214062 China; 5 The MOE Key Laboratory for Standardization of Chinese Medicines, Institute of Chinese Materia Medica, Shanghai University of Traditional Chinese Mdicine, Shanghai, 201203 China

## Correction:

 *Mol Cancer* **19**, 51 (2020)

10.1186/s12943-020-01174-w

Following publication of the original article [1], the authors identified an error in Fig. 8. In the version of this article initially published, they found one panel in Fig. 8d was used by mistake. The authors want to update it to the correct one. The other elements of the figure remain the same, and the interpretation of the results remains unchanged. The authors’ subsequent studies on MIR22HG have confirmed that the results and conclusions in this study are correct and not affected by this erratum. The correct figure is given below.

**Fig. 8:**
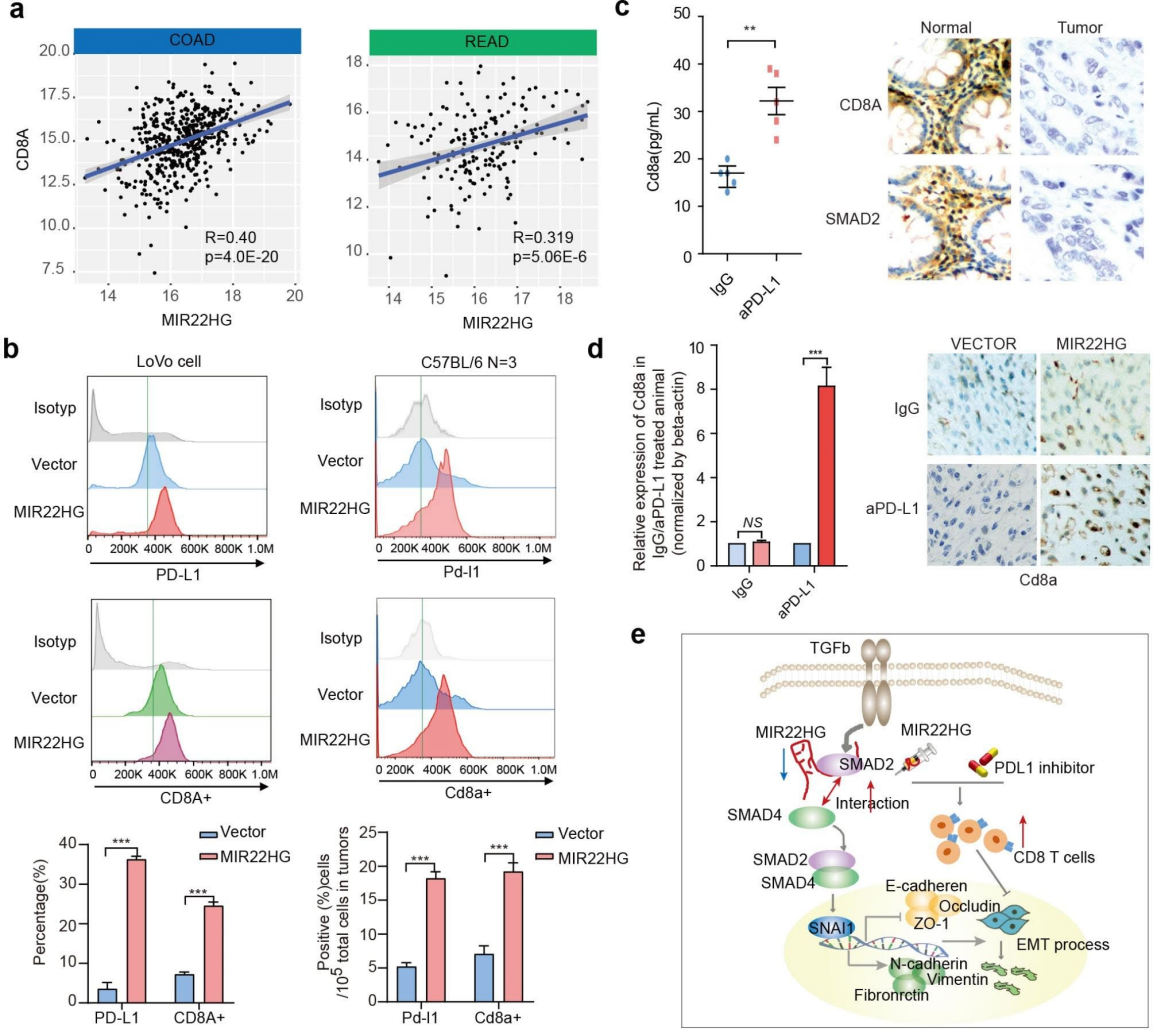
MIR22HG increases the CD8 T cells in CRC. **a**, Scatter plots showing the correlation between CD8A expression and MIR22HG expression. **b**, Cell-surface expression of PD-L1 and CD8A with overexpressing MIR22HG. Left panels for human cell line and right panels for mouse. **c**, Relative expression of Cd8a in mice treated with aPD-L1. The right panels showing the IHC staining of CD8A and SMAD2 in tumor and normal tissues of CRC. **d**, Left panel showing the relative expression of Cd8a in IgG/aPD-L1 + MIR22HG treated mice. Right panel showing the IHC staining of Cd8a. **e**, The mechanistic scheme of lncRNA MIR22HG in CRC
