# Supplementary material for: MIR22HG acts as a tumor suppressor via TGFβ/SMAD signaling and facilitates immunotherapy in colorectal cancer
Source: Mol Cancer. 2020 Mar 4;19:51. doi: 10.1186/s12943-020-01174-w (PMC7055097; doi:10.1186/s12943-020-01174-w)
Supplement: Supplementary file 1 — Additional file 1. Supporting Materials and Methods. [file 12943_2020_1174_MOESM1_ESM.docx]

**Supporting Materials and Methods**

**Quantitative RT-PCR**

Total RNA was reverse transcribed into cDNA using the Prime-Script II 1st Strand Synthesis Kit (TaKaRa). Quantitative RT-PCR (qRT-PCR) was performed on the ViiA7 real-time PCR system using the UltraSYBR Mixture (CWBIO, China). The relative gene expression levels was normalized to those of β-actin and calculated based on 2^-△△Ct^.

**Vector construction and siRNA**

The MIR22HG sequence was synthesized by GENEray Biotechnology (China) and cloned into the eukaryotic expression vector pcDNA3.1 and the lentivirus expression vector pWPXL. The siRNAs of MIR22HG and SMDA2 were purchased from RiboBio (China). The validated shRNA sequence of MIR22HG was synthesized and cloned into the shRNA cloning and expression lentivector.

**MIR22HG overexpression or knockdown**

The pWPXL, pWPXL-MIR22HG, pWPXL-shMIR22HG plasmid was co-transfected into HEK-293T cells along with the packaging plasmid ps-PAX2 and the envelope plasmid pMD2G using Lipofectamine 2000 (Invitrogen) as previously described [1]. The virus particles were harvested 48 hours after co-transfection and then individually used to infect colorectal cancer cells to generate corresponding stable cell lines. The efficiency of MIR22HG overexpression or knockdown was assessed by qRT-PCR.

**Cell proliferation and colony formation assay**

Cell viability was measured using the Cell Counting Kit 8 (CCK-8, Dojindo, Japan) according to the manufacturer's instructions. For the colony formation assay, 500 to 1,000 colorectal cancer cells were seeded into each well of a 6-well plate and maintained in a medium containing 10% FBS for 15 days. The colonies were fixed with methanol and stained with 0.1% crystal violet. The number of clones was counted using an inverted microscope.

**Cell migration and invasion assays**

Cell migration and invasion assays were performed using Boyden Transwell chambers (8-mm pore size, BD Biosciences) as previously described [1].

**Tumor formation and metastasis assays in nude mouse models**

LoVo cells stably expressing MIR22HG or the control vector were subcutaneously injected into either flank of the same athymic male nude mouse at 6 weeks of age (n=6 for each group). Four to 6 weeks after injection, the mice were sacrificed and examined for the growth of subcutaneous tumors. Two types of mouse models were used to evaluate the effect of MIR22HG on colorectal cancer metastasis. For the in vivo lung metastasis assay, 2 x10^6^ LoVo cells stably expressing MIR22HG or the control vector were injected into the inferior mesenteric of each nude mouse (n=10 for each group). These mice were sacrificed after 6 weeks and examined for lung metastases. In addition, an orthotopic mouse model was used to evaluate the effect of MIR22HG on hepatic metastasis. All animal care and handling procedures were performed in accordance with the National Institutes of Health's Guide for the Care and Use of Laboratory Animals [2]. All animal experiments were approved by the Clinical Research Ethics Committees of Affiliated Hospital of Jiangnan University

**RNA pull-down assays and mass spectrometry analyses**

RNA pull-down assays were performed using the Pierce Magnetic RNA-Protein Pull-Down Kit (Thermo Fisher) according to the manufacturer's instructions. Briefly, the MIR22HG sequence was in vitro transcribed with biotin RNA-labeling mix and T7 RNA polymerase (Invitrogen) according to the manufacturer's instructions. The biotinylated MIR22HG RNA was incubated with streptavidin-linked magnetic beads and total cell lysates at room temperature for 2 hours. The bead-RNA–protein complexes were washed with 1x binding washing buffer four times. The proteins were precipitated and diluted in protein lysis buffer. Finally, the retrieved proteins were measured on SDS-PAGE gels for one-shot mass spectrometry or Western blot analysis. Detailed information regarding the primers used for in vitro transcription is TAATACGACTCACTATAG.

**RNA Immunoprecipitation assay**

RNA Immunoprecipitation (RIP) assays were performed using the Magna RIP RNA-Binding Protein Immunoprecipitation Kit (Millipore). Briefly, 1x10^7^ colorectal cancer cells were harvested and lysed with RIP lysis buffer. Cell extracts were co-immunoprecipitated with anti-SMAD2 antibodies (CST), and the retrieved RNA was subjected to qRT-PCR analysis using MIR22HG-specific primers. Total RNA (input controls) and normal mouse IgG controls were assayed simultaneously to confirm that the detected signals were from the RNA specifically binding to SMAD2.

**Western blotting**

The separation of nuclear and cytoplasmic fractions was performed using the PARIS Kit (Life Technologies) according to the manufacturer's instructions. Extracted proteins were separated by SDS-PAGE and transferred to a PVDF membrane. The membrane was blocked with 5% non-fat milk and incubated with primary antibodies for SMAD2 (CST, 1:1,000), SMAD4 (CST, 1:1,000), β-Actin (CST;4970 1:10000), N-Cadherin (CST,1:500), E-Cadherin (CST,1:400), ZO-1 (CST,1:500), Vimentin (AV48225), Occludin (Sigma ,1:500), Cd8a (Sigma 1:500) and Fibronectin 1 (Sigma,1:500).

**Immunohistochemistry**

The expression levels of SMAD2, PD-L1 protein were determined by immunohistochemistry (IHC) analysis. IHC staining was performed on 4-mm sections of paraffin-embedded tissue samples. Briefly, the slides were incubated with a pd-l1 (abcam 1:200) or SMAD2 antibody (CST, 1:200) at 4°C overnight. The subsequent steps were performed using the GTVision III Detection System/Mo&Rb (Gene Tech, China).

**Antibodies and reagents**

Anti-SMAD2 (5339), Anti-β-Actin (4970), Anti-SMAD2 (46535), Anti-N-Cadherin (99377), Anti-E-Cadherin (14472), Anti-ZO-1 (14472), Smad2 (8685) antibodies were purchase from Cell Signaling Technology. Anti-Vimentin (AV48225), Anti-Occludin (SAB4200593), Anti-Cd8a (SAB4700563) and Anti-Fibronectin 1 (SAB4500974) antibodies were purchase from Sigma-Aldrich. Anti-pd-l1 (ab233482), Anti-IgG (ab6789), antibodies were purchase from abcam. Isotype control IgG and anti-PD-L1 (BE0101, clone 10F.9G2) antibodies were purchased from Bio X Cell. The ELISA kits of IgG and PD-L1 were purchase from Thermofisher.

**RNA interference**

Knockdown was achieved by RNA interference. The shRNA sequences were as follows: TCCAGTAGAAGGCTCGCGCTTGG and ATCCGTAGCCGCTCAAACTCTCC. The shRNA sequences were as follows: mouse *Mir22hg*: TCGATGGTTACGCGTGTTGA; TTGCTATGTTAGGACCTTCA.

The shRNA sequences for SMAD2 were

siSMAD2-1:GUCCCAUGAAAAGACUUAA;

siSMAD2-2:UCUUUGUGCAGAGCCCCAA;

siSMAD2-3:GCUUAGGUUUACUCUCCAAUG;

siRNA transfection was carried out using Lipofectamine 2000 reagent (Invitrogen Co.) according to the manufacturer's instructions.

**Immunoblotting and Immunofluorescence**

Cells were washed in PBS, and cellular proteins were extracted in 10 mol/L urea lysis buffer plus protease and phosphatase inhibitors (GenDEPOT) for 50 min at 4°C. Lysates were cleared by centrifugation, and proteins were separated by gel electrophoresis. Membranes were blocked in PBS 0.1% Tween 20 (PBS-T)/5% (w/v) milk for 1hr at room temperature. Membranes were then incubated with primary antibodies diluted in PBS-T/5% (w/v) milk at 4°C overnight. Subsequently, membranes were washed with PBS-T and incubated with horseradish peroxidase secondary antibody (1:2,000; Jackson ImmunoResearch) diluted in PBS-T/5% skim milk. Membranes were washed in PBS-T, and bound antibody was detected by enhanced chemiluminescence (GE Healthcare).

**ELISA**

The cell culture supernatant or ascites from ID8 model were collected and processed according to the manufacturer’s instructions [3]. The Cd8a levels were determined using ELISA kits from R&D/Thermo Fisher Scientific following the standard procedures.

**Colorectal Cancer BALB/C Model**

Mouse experiments were conducted as described previously. Briefly, murine colorectal cancer cells LoVo (2 x 10^6^) were subcutaneously injected into the left flank of athymic mice (5–7 weeks old, CRL/NCI). Mice were allowed to recover and monitored closely for the next 24 hours. Tumor size was measured every three days and the tumor volume was determined based on the calculation (width × width × length)/2. Tumor bearing mice were treated (i.p.) with isotype control IgG or anti-PD-L1 antibody (200 μg/mouse) every three days. Mice reaching an endpoint requiring euthanasia by IACUC guidelines or exceeding tumor burden limits were euthanized.

**Flow cytometer**

The antibodies were purchased from Biolegend: PD-L1 (329718), CD8A (301008), anti-mouse IgG2b Antibody isotype (406708), anti-Mouse IgG1 isotype (406608), the cell surfaces were stained by a standard protocol. Stained cells were isolated by flow cytometry and the results analyzed by FlowJo software.

Colony tumor cells LoVo (1x10^6^ per mouse) were subcutaneously inoculated into C57BL/6 mice. After 20 days primary tumors were surgically removed after inoculation. A sample of each tumor from each treatment group was dissociated with Liberase TM (Sigma-Aldrich) enzyme cocktail. Cells were sequentially incubated with primary and secondary antibodies for 30 minutes each at 4°C. Stained cells were analyzed on an Attune acoustic focusing cytometer (Applied Biosystems). Flow cytometry data were analyzed by the FlowJo software program (BD Biosciences).

LoVo cell were stained with the following antibodies: FITC-conjugated anti-PD-L1, PE-conjugated anti-CD8. Samples were analyzed using a BD FACS ARIA (BD Biosciences).

**Cell Co-culture**

Peripheral blood mononuclear cells (PBMC) were isolated with Ficoll–Hypaque by density gradient centrifugation within 2 hours of sample collection from Healthy donor. Cells were grown in RPMI1640 medium supplemented with 10% FBS, 50 U/mL penicillin, and 50 mg/mL streptomycin in a humidified atmosphere at 37°C with 5% CO2. PBMC cells were sorted and co-cultured with colon cancer cell line LoVo cells in 48-well plates at a ratio of 5:1. After 48h we collected the cell for flow cytometry.

**Inhibition of TGF-beta signaling pathway**

Galunisertib (LY-2157299 monohydrate) is an oral small molecule inhibitor of the TGF-β receptor I kinase that specifically down-regulates the phosphorylation of SMAD2, abrogating activation of the canonical pathway [4, 5]. 2μM galunisertib was added with DMSO as vehicle control. Cell proliferation and migration were measured by CCK8 and tranwell as above after 3 days of culture. Western blot and qPCR was measured as above after 2 days of culture.

**References**

1. Zhang J, Li Z, Liu L, Wang Q, Li S, Chen D, Hu Z, Yu T, Ding J, Li J, et al: **Long noncoding RNA TSLNC8 is a tumor suppressor that inactivates the interleukin-6/STAT3 signaling pathway.** *Hepatology* 2018, **67:**171-187.

2. In *Guide for the Care and Use of Laboratory Animals.* Edited by th. Washington (DC)2011: *The National Academies Collection: Reports funded by National Institutes of Health*].

3. Duraiswamy J, Freeman GJ, Coukos G: **Therapeutic PD-1 pathway blockade augments with other modalities of immunotherapy T-cell function to prevent immune decline in ovarian cancer.** *Cancer Res* 2013, **73:**6900-6912.

4. Zhou L, McMahon C, Bhagat T, Alencar C, Yu Y, Fazzari M, Sohal D, Heuck C, Gundabolu K, Ng C, et al: **Reduced SMAD7 leads to overactivation of TGF-beta signaling in MDS that can be reversed by a specific inhibitor of TGF-beta receptor I kinase.** *Cancer Res* 2011, **71:**955-963.

5. Bueno L, de Alwis DP, Pitou C, Yingling J, Lahn M, Glatt S, Troconiz IF: **Semi-mechanistic modelling of the tumour growth inhibitory effects of LY2157299, a new type I receptor TGF-beta kinase antagonist, in mice.** *Eur J Cancer* 2008, **44:**142-150.
