# Supplementary material for: MIR22HG acts as a tumor suppressor via TGFβ/SMAD signaling and facilitates immunotherapy in colorectal cancer
Source: Mol Cancer. 2020 Mar 4;19:51. doi: 10.1186/s12943-020-01174-w (PMC7055097; doi:10.1186/s12943-020-01174-w)
Supplement: Supplementary file 2 — Additional file 2. Supplementary figures, including supplementary Figs S1-S10. [file 12943_2020_1174_MOESM2_ESM.docx]

**
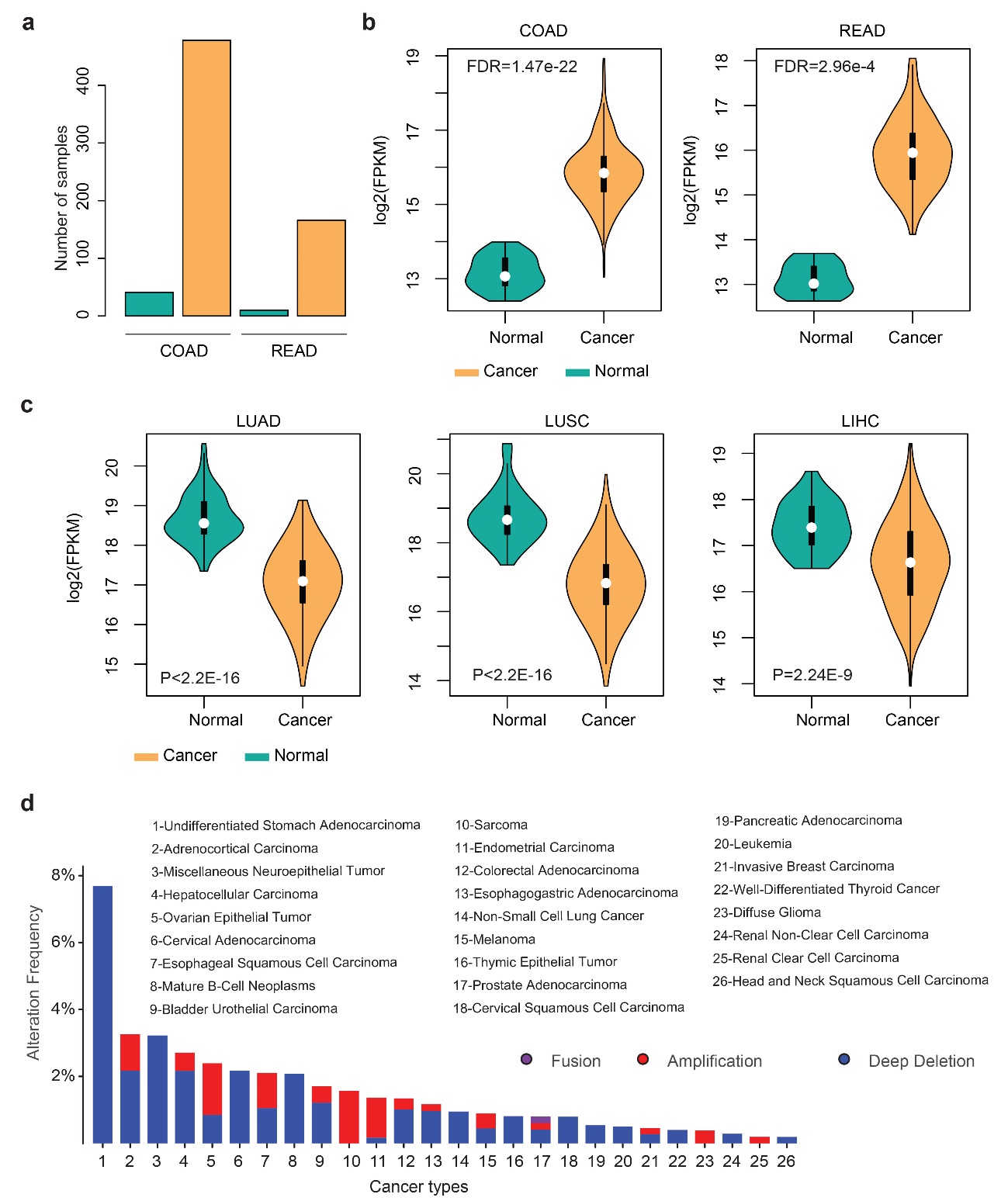
**

**Fig. S1. The expression of MIR22HG in cancer.** **a**, The number of tumor and normal samples in COAD and READ. **b**, The expression distribution of PVT1 in tumor and normal samples. Left is for COAD and right is for READ. **c**, The expression distribution of MIR22HG in LUAD, LUSC and LIHC. **d**, The copy number variation and fusion alteration frequency of MIR22HG across cancer types.

**
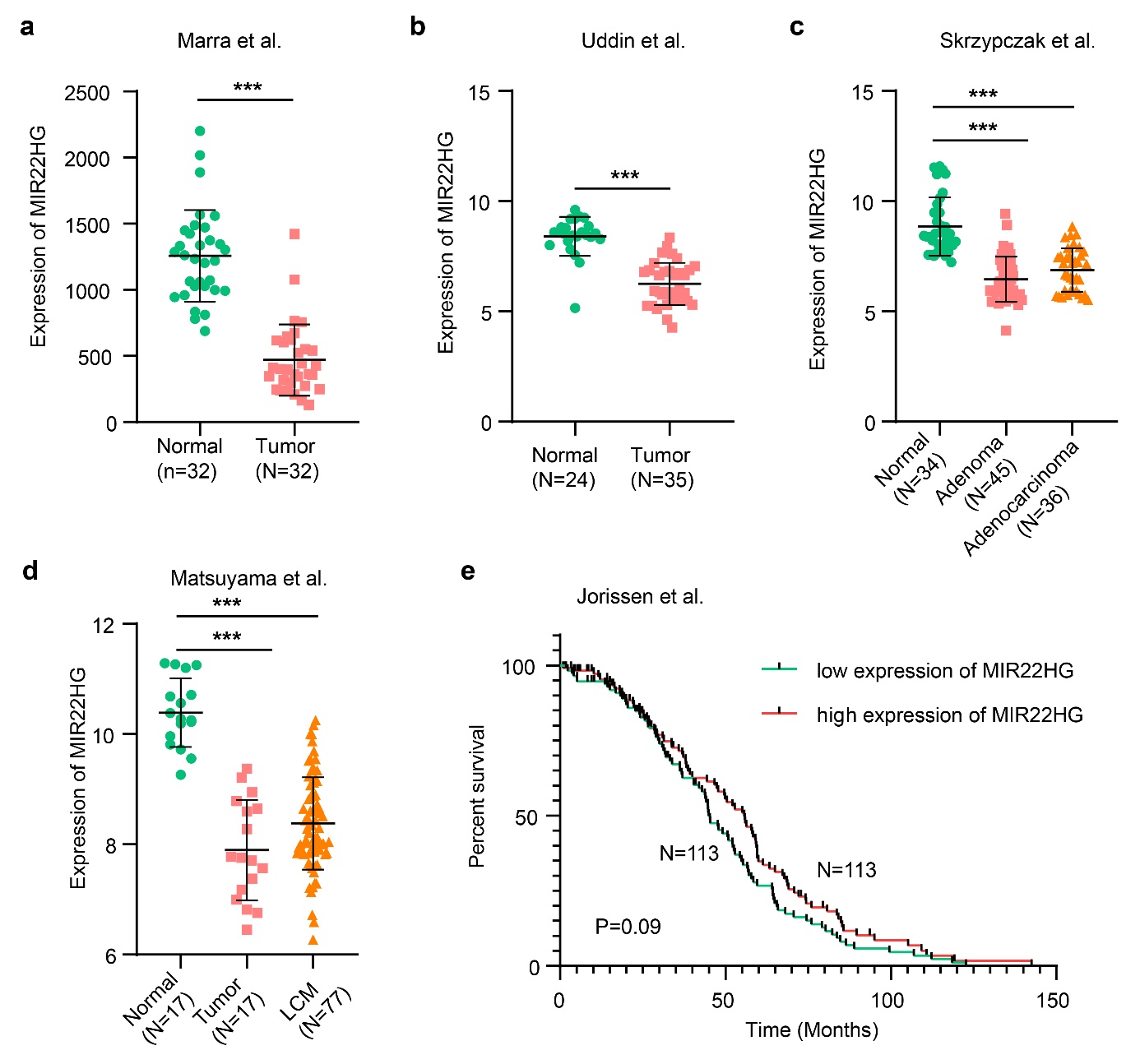
**

**Fig. S2. Validation of MIR22HG expression in independent datasets.** **a-d**, The expression distribution of MIR22HG in four independent datasets. **e**, Kaplan-Meier overall survival of CRC patients based on expression of MIR22HG.


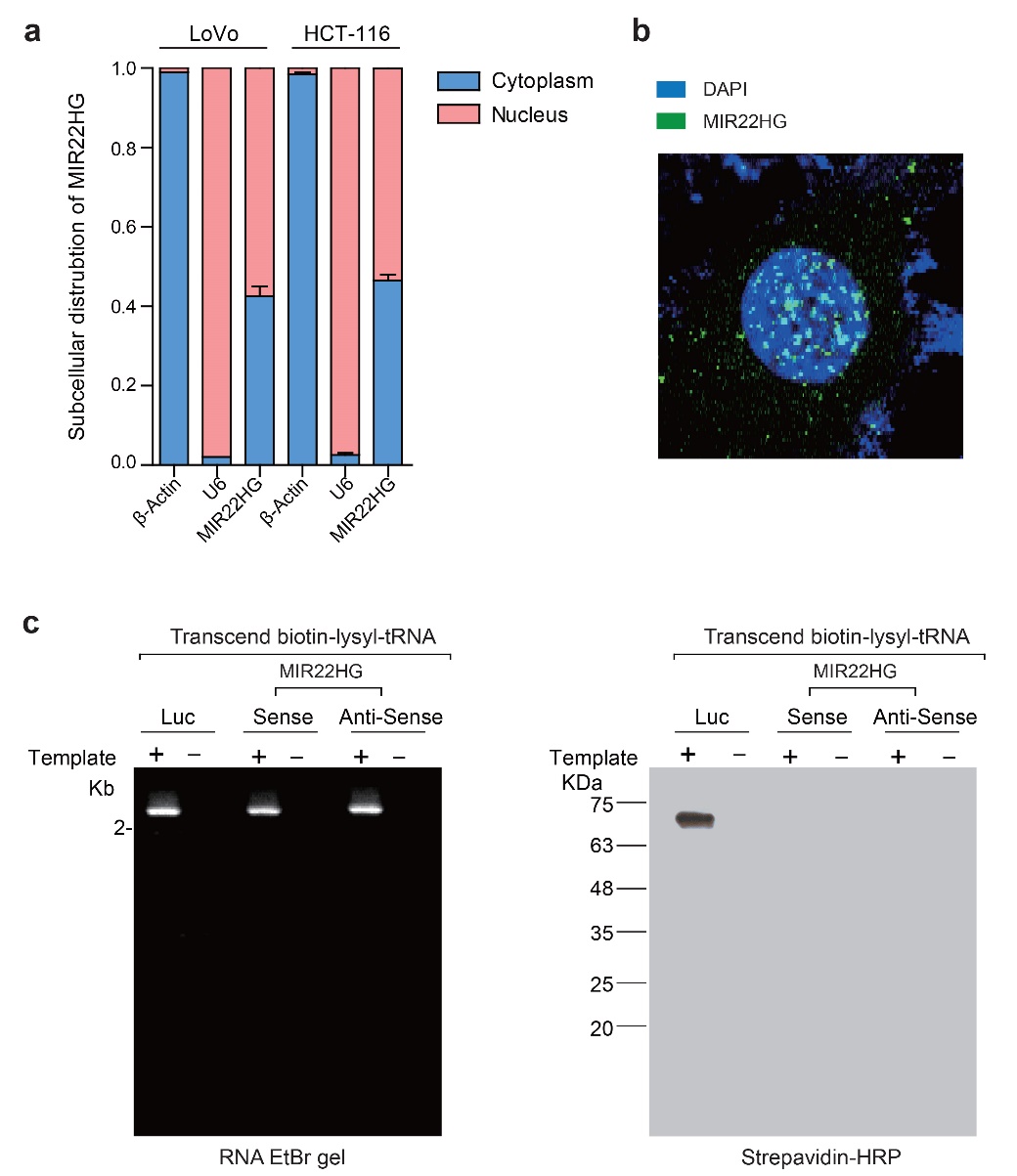


**Fig. S3. Subcellular location of MIR22HG.** **a**, The proportion of MIR22HG localized in cytoplasm and nucleus in two cell lines. β-Actin served as the cytoplasmic internal control. U6 served as the nuclear internal control. **b**, Representative image for MIR22HG location in the HCT-116 colon cancer cell. **c**, In vitro transcription and translation of MIR22HG sense or antisense transcript. Luciferase (Luc) is used as a positive control.


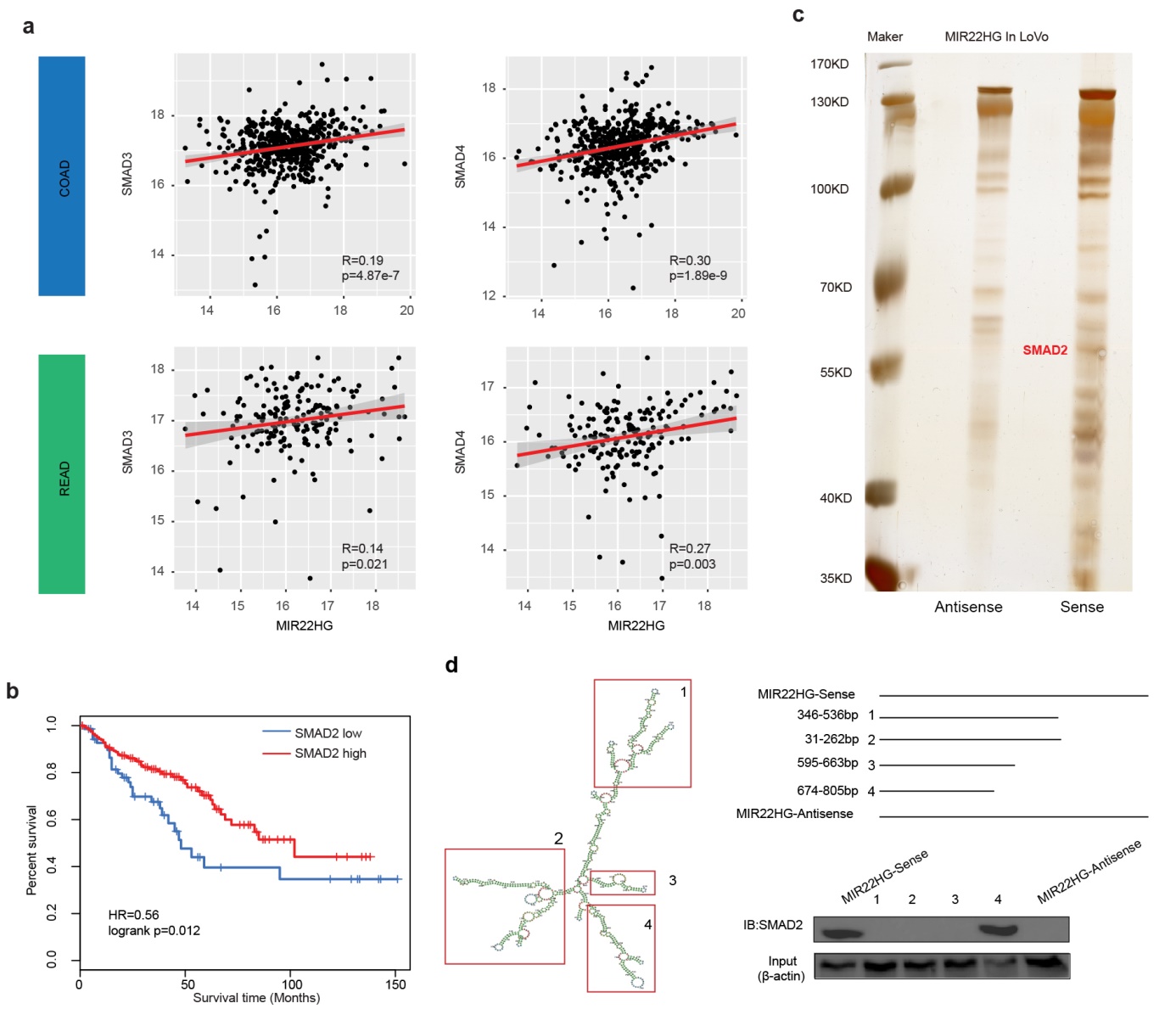


**Fig. S4. Interaction between MIR22HG and SMADs in CRC.** **a**, Scatter plots showing the correlation between expression of SMAD3/4 and MIR22HG. **b**, KM plot showing the association of SMAD2 expression with patient survival. **c**, Proteins retrieved from the MIR22HG RNA pull-down assay were analyzed by SDS-PAGE. **d**, Immunoblot detection of the SMAD2 protein in LoVo cells as retrieved by in vitro transcribed biotinylated RNAs of different constructs of MIR22HG or its antisense sequence (negative control). Left panel showing the secondary structure of MIR22HG analyzed by LNCipedia.

**
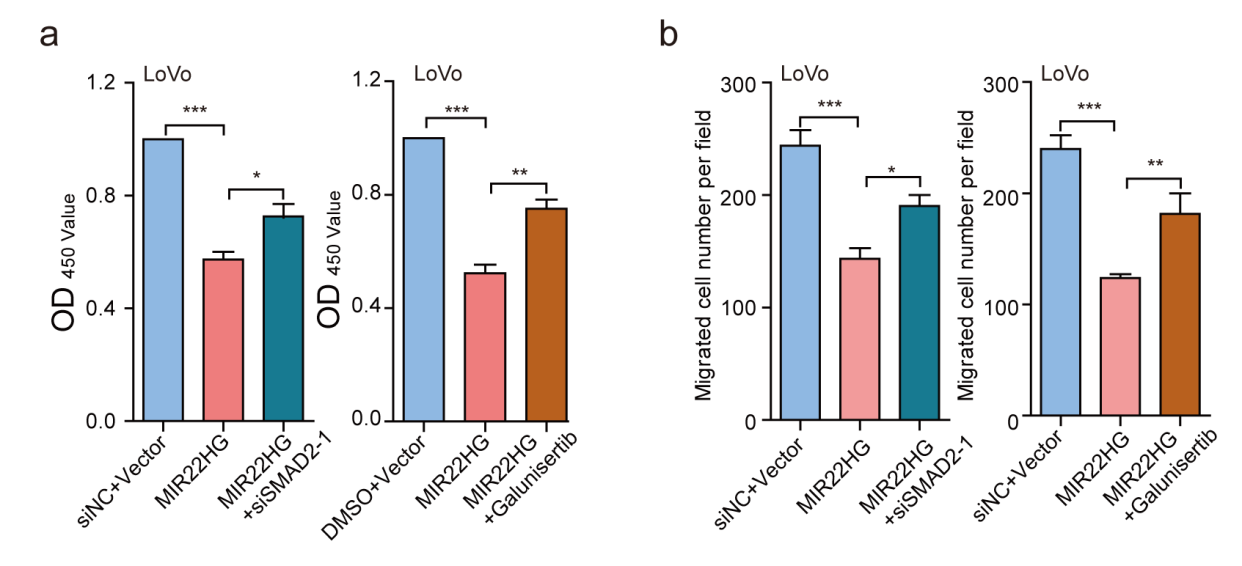
**

**Fig. S5. MIR22HG exerts its biological function of inhibiting the growth and migration of colorectal cancer cells through SMAD2. a,** Interfering the expression of SMAD2 and activity of TGFβ pathway reverse the biological function of MIR22GH inhibiting the proliferation of colorectal cancer cells. **b,** Interfering the expression of SMAD2 and activity of TGFβ pathway reverse the biological function of MIR22GH inhibiting colorectal cancer cell migration.


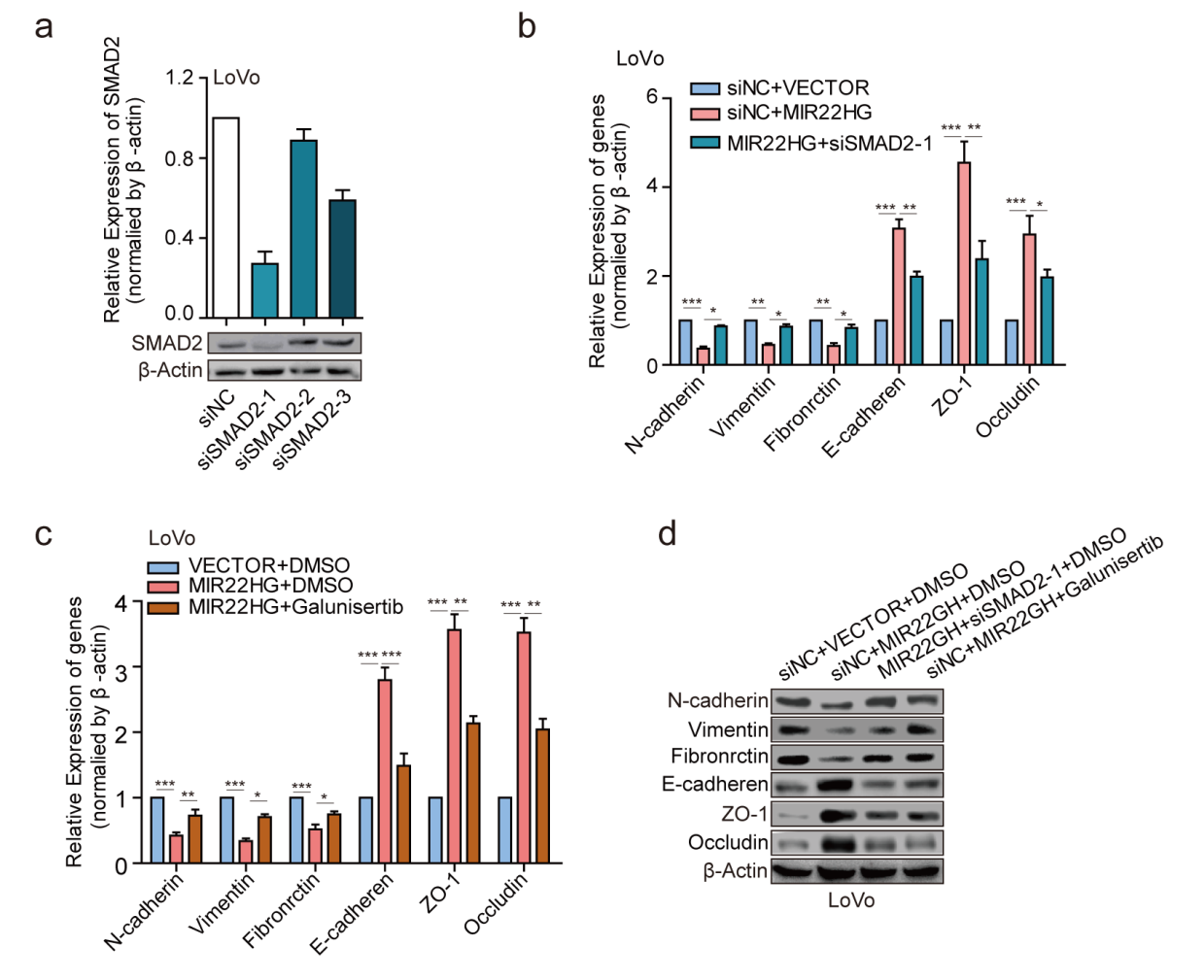


**Fig. S6. MIR22HG affects TGF-β signaling pathway and inhibits EMT through SMAD2.** **a**, Verify siSMAD2 interference efficiency with qPCR and Western-blot. **b**, qPCR showing the expression of EMT genes, suggesting that interference SMAD2 reverses MIR22HG inhibition of EMT. **c**, qPCR showing the expression of EMT genes when TNFβ signaling pathway was inhibited. **d**, Western-blot shows the expression of EMT proteins interferes with SMDA2 expression and TNFβ signaling pathway inhibitor and MIR22HG.

**
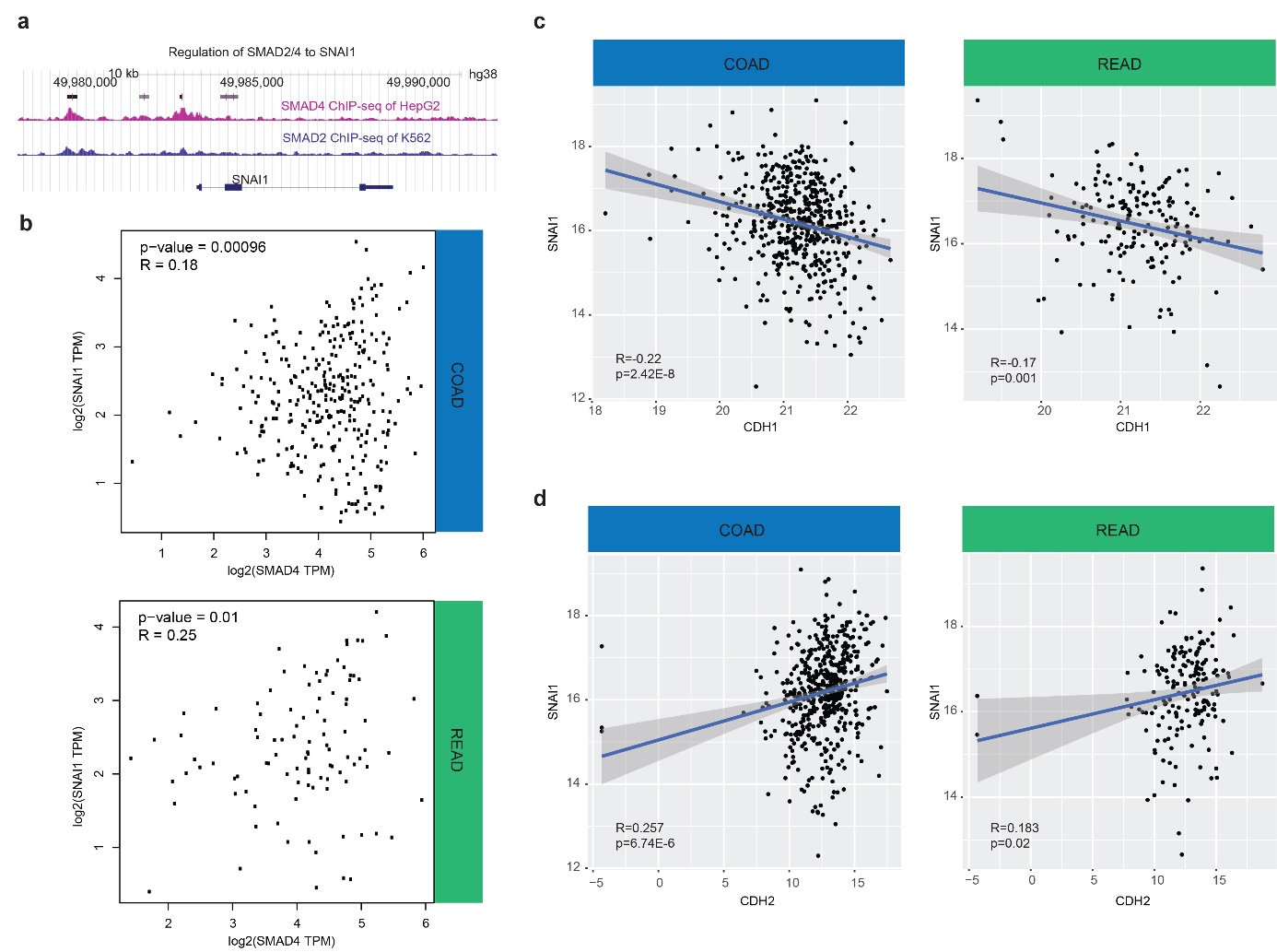
**

**Fig. S7. Regulation between SMADs-SNAI1-EMT axis.** **a**, The reads distribution of SMAD2/SAMD4 around the promoter of SNAI1 in HepG2 cell line. **b**, The scatter plots showing expression correlation between SMAD4 and SNAI1 in COAD and READ. **c**, The scatter plots showing expression correlation between SNAI1 and CDH1 in COAD and READ. **d**, The scatter plots showing expression correlation between SNAI1 and CDH2 in COAD and READ.


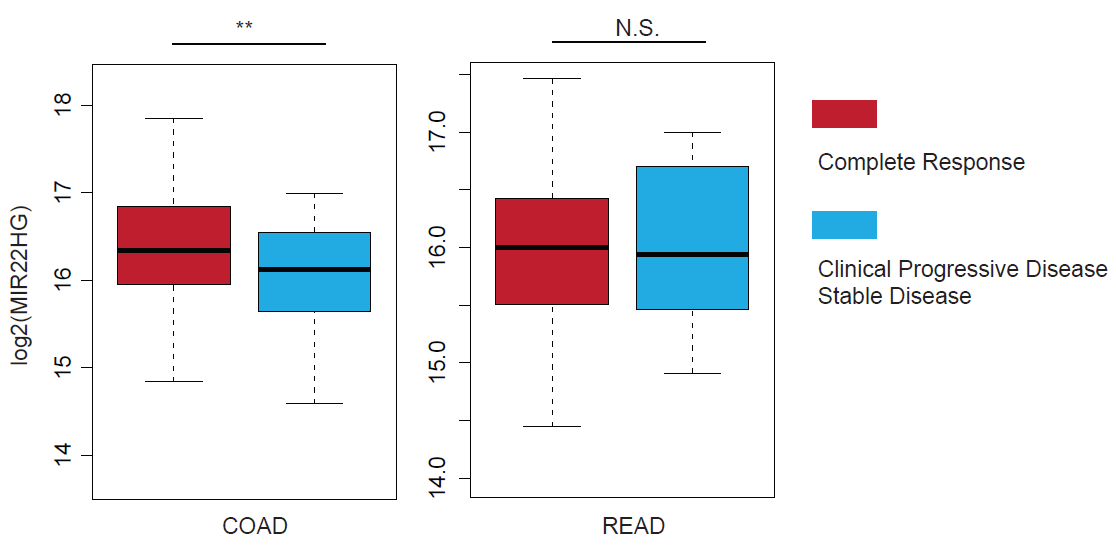


**Fig. S8. Distribution of MIR22HG expression in patients with different response for drug treatment**. Left panel is for COAD patients and right panel is for READ patients. **, p<0.05.


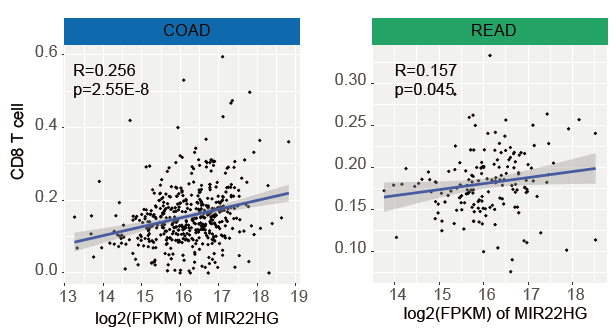


**Fig. S9. Scatter plots showing the correlation between expression of MIR22HG and CD8 T cell infiltration**. Left panel is for COAD patients and right panel is for READ patients.


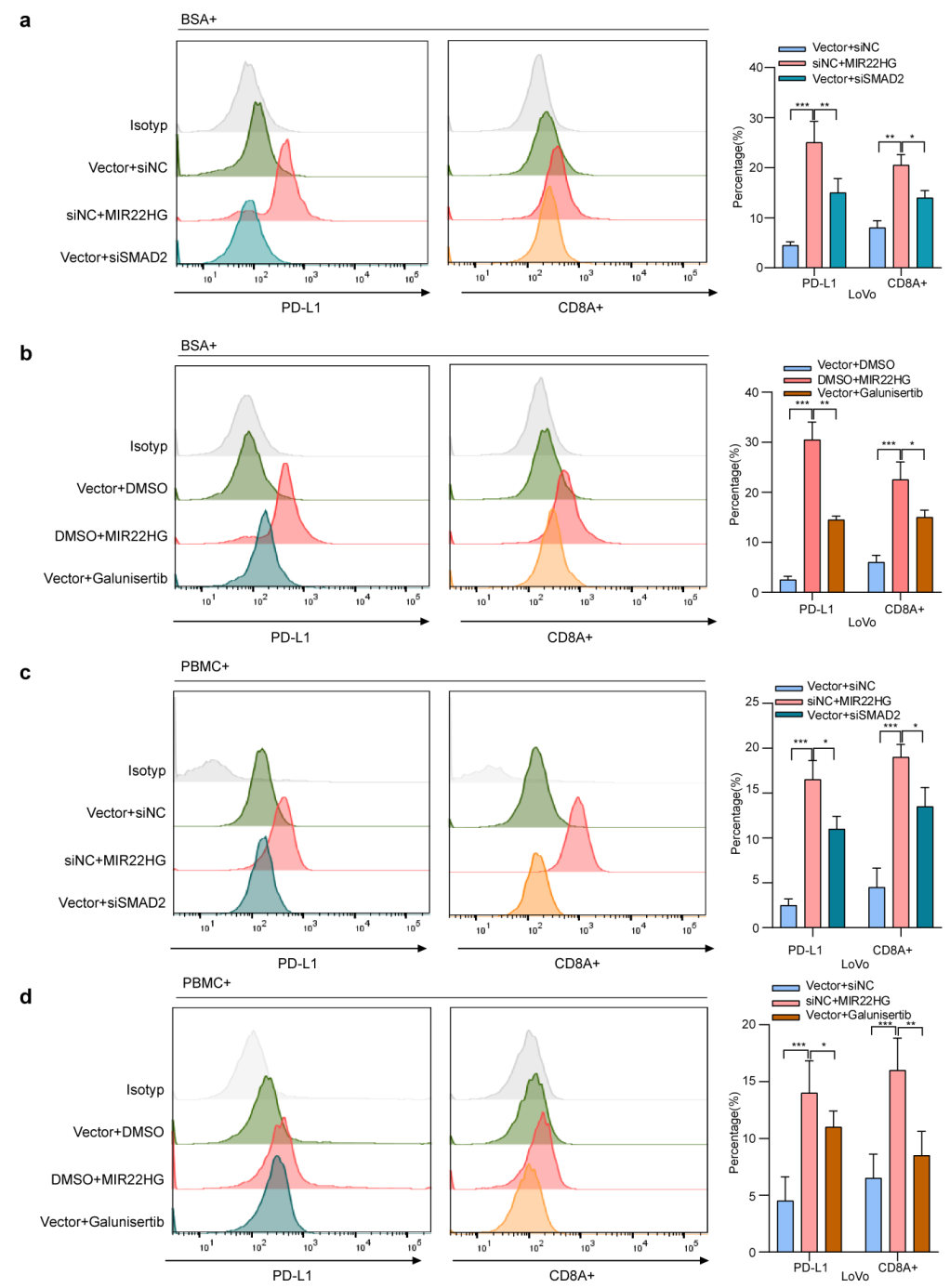


**Fig. S10. MIR22HG affects the expression of CD8 and PD-L1 through SMAD2 affecting the TGF-β signaling pathway. a,** Interference SMAD2 expression reversed MIR22HG promotes CD8 and PD-L1 expression by flow cytometry. **b,** TGF-β inhibitor reversed MIR22HG promotes CD8 and PD-L1 expression Flow cytometry. **c,** Interference SMAD2 expression reversed the expression of CD8 and PD-L1 by MIR22HG under PBMCs co-cultured with LoVo by flow cytometry. **d,** TGF-β inhibitors reversed MIR22HG promotes CD8 and PD-L1 expression under PBMCs co-cultured with LoVo by flow cytometry.
