# Supplementary material for: MIR22HG acts as a tumor suppressor via TGFβ/SMAD signaling and facilitates immunotherapy in colorectal cancer
Source: Mol Cancer. 2020 Mar 4;19:51. doi: 10.1186/s12943-020-01174-w (PMC7055097; doi:10.1186/s12943-020-01174-w)
Supplement: Supplementary file 3 — Additional file 3. Table S1. Correlation of the expression of MIR22HG in colorectal cancer with clinic pathologic features. [file 12943_2020_1174_MOESM3_ESM.docx]

**Table S1. Correlation of the expression of MIR22HG in colorectal cancer with clinic pathologic features.**

| **Parameter** | **No. of patients** | **MIR22HG (low)** | **MIR22HG (high)** | ***P* -value** |
| --- | --- | --- | --- | --- |
| **Sex** |  |  |  | 0.434 |
| male | 87 | 46 | 41 |  |
| female | 76 | 35 | 41 |  |
| **Age (yr)** |  |  |  | 0.527 |
| < 60 | 68 | 36 | 32 |  |
| ≥ 60 | 95 | 45 | 50 |  |
| **Tumor size (cm)** | |  |  | **0.0001** |
| <5 | 89 | 24 | 65 |  |
| ≥ 5 | 74 | 40 | 34 |  |
| **Location** | |  |  | 0. 873 |
| Colon | 66 | 36 | 30 |  |
| Rectum | 97 | 51 | 46 |  |
| **Differentiation** | |  |  | **<0.0001** |
| Well and moderately | 78 | 23 | 55 |  |
| Poorly | 85 | 60 | 25 |  |
| **Depth of tumor** | |  |  | **0.0001** |
| T1 +T2 | 67 | 24 | 43 |  |
| T3+T4 | 98 | 62 | 36 |  |
| **Differentiation grade** |  |  |  | 1.000 |
| I-II | 55 | 23 | 32 |  |
| III-IV | 108 | 45 | 63 |  |
|  |  |  |  |  |
